# A solution right under our nose? Crossbreeding extreme brachycephalic dogs results in better health outcomes while preserving behavioural profiles and dog-owner relationship quality

**DOI:** 10.1017/awf.2026.10095

**Published:** 2026-08-05

**Authors:** Elizabeth Youens, Dan G O’Neill, Mickey Tivers, Zoe Belshaw, Johanna Neufuss, Rowena M. A. Packer

**Affiliations:** 1 Clinical Science and Services, Royal Veterinary College, Herts, UK; 2 Pathobiology and Population Sciences, Royal Veterinary College, Herts, UK; 3 Paragon Veterinary Referrals, Wakefield, UK; 4 EviVet Evidence-Based Veterinary Consultancy, Nottingham, UK; 5 Blue Cross, Burford, UK

**Keywords:** Animal welfare, breeding, canine, conformation, crossbreed, outcross

## Abstract

Despite substantial evidence of numerous welfare-limiting disorder predispositions linked to their exaggerated conformation, brachycephalic dogs remain highly desired breeds in the UK. National and international attempts to reduce demand for brachycephalic breeds appear to have had a limited effect on significantly reducing their acquisition. As ‘designer crossbreeds’ become increasingly popular, an opportunity exists to leverage the public’s desire for intentionally crossbred dogs to meet the ownership niche currently occupied by extreme brachycephalic dogs. However, evidence on the comparative health, behaviour and dog-owner relationship between extreme brachycephalic purebreds and their less extreme designer crosses is urgently needed to understand whether they are truly higher-welfare. Purebred Pug (n = 51) and Pug-cross (‘Jug’: Jack Russell × Pug; n = 51; and ‘Puggle’: Beagle × Pug; n = 48) dogs were recruited from the UK pet-owned population for veterinary health assessments and to assess owner-reported health, behaviour and dog-owner relationships using established tools. Jugs and Puggles had a less extreme facial conformation than Pugs, with significantly longer relative muzzle length and less stenotic nostrils. Purebred Pugs exhibited a significantly higher prevalence of respiratory, ocular, dermatological and musculoskeletal disorders compared to Jugs and Puggles, and accrued increased veterinary costs. However, behaviour profiles and dog-owner relationships were similar between purebred and designer crossbred dogs. These results provide strong evidence of substantially reduced conformation-related disorder burden in designer crossbreed Pugs compared to their purebred Pug progenitor, whilst preserving behaviour profiles and dog-owner relationships that are known key motivators for extreme brachycephalic acquisition. Crossbreeding offers major opportunities to improve the welfare of extreme brachycephalic dogs.

## Introduction

Brachycephaly in dogs describes a flat-faced (short-muzzled) appearance that results from active selection for multiple mutations causing foreshortening of the facial skeleton (Ekenstedt *et al.* 2020). This phenotype has been rigorously and rapidly selected for in certain dog breeds, such as Pugs, English Bulldogs and French Bulldogs. Drivers for this extreme conformation include breed standards published by breeding organisations, such as the Royal Kennel Club (Royal Kennel Club 2025a) and prolonged inbreeding (mating between closely related individuals), which continues to be widely accepted as critical to develop and then maintain pedigree ‘purity’ (Packer *et al.* 2015a,b; Pederson *et al.* 2016; Worboys *et al.* 2018). Unfortunately for the affected dogs, brachycephaly is associated with substantial welfare compromise through multiple conformation-related disorders including, but not limited to, Brachycephalic Obstructive Airway Syndrome (BOAS) (Packer *et al.* 2015b; O’Neill *et al.* 2015), ocular (Packer *et al.* 2015a; O’Neill *et al.* 2017a, 2021a, 2022c), dermatological (O’Neill *et al.* 2022a), reproductive (O’Neill *et al.* 2017b), spinal (Ryan *et al.* 2017, 2019), and heat-related illness (Hall *et al.* 2020).

Breeding towards less extreme conformations could reduce the welfare harms for brachycephalic dogs. Focus to date has been on improving BOAS over other conformation-related disorders. For the Pug, French Bulldog and English Bulldog, the dominant health scheme proposed for reducing incidence and severity of BOAS is the respiratory functional grading scheme (RFGS) (Liu *et al.* 2015), adopted as an exercise-based grading tool by the Royal Kennel Club (Royal Kennel Club 2026). The RFGS aims to offer owners information on the severity of their dog’s respiratory dysfunction, supports monitoring of disease, aids in surgical assessment and is also suggested to guide breeders on risks of breeding affected puppies based on their parents’ gradings (Ladlow 2021). However, given the plethora of other serious conformation-related disorders documented in extreme brachycephalic dogs, root-cause approaches targeting conformation directly are likely needed for major welfare improvements.

An alternative strategy is to purposefully breed towards dogs with less extreme conformation. Brachycephaly is a continuous rather than a binary trait, with relative muzzle length to cranial length (craniofacial ratio; CFR) varying between brachycephalic breeds and also between individual dogs within these breeds (Packer *et al.* 2015b). The risk of BOAS increases as CFR shortens (Packer *et al.* 2015b; Tomlinson *et al.* 2025, 2026). Facial conformation, including eyelid aperture size and the presence of nasal folds also increases the risk of other conformation-related disorders, such as corneal ulceration (Packer *et al.* 2015b). Therefore, breeding towards conformations with relatively longer craniofacial ratios, reduced relative eyelid aperture and reduced or absent nasal folds, alongside owners maintaining an ideal body condition score in their dogs, could reasonably be expected to reduce risk of conformational-related disorders within affected breeds (Packer *et al.* 2015a,b). However, phenotypic variability within existing purebred populations of some brachycephalic breeds is severely restricted towards extreme conformations (Packer *et al.* 2015a,b), limiting the scope for within-breed selection to move towards the necessary less-extreme body shapes. Furthermore, despite veterinary surgeons considering active alteration of the breed standard as key action to improve breed health (Farrow *et al.* 2014), there is substantial resistance to major changes to breed standards from the pedigree dog breeding sector. A recent Swedish study reported a wide disconnect between show judges and other stakeholders, with judges proposing it was essential to follow breed standards even when health is negatively affected, whilst owners, breeders and veterinary surgeons disagreed (Åsbjer *et al.* 2024).

More widely, reduction in ownership of brachycephalic dogs is seen as critical to protect dog welfare at a population level and has been promoted in various public-facing UK campaigns (RSPCA 2011, 2024; BVA 2018; Blue Cross 2021; UK-BWG 2021a; ICECDogs 2024). Although there are some indications of waning ownership demand for brachycephalic dogs in the UK (Royal Kennel Club 2025b), extreme breeds such as the French Bulldog remain in the top five most registered breeds by the Royal Kennel Club (Royal Kennel Club 2025c), and French Bulldogs and Pugs have been reported as the second and third most advertised breeds on the selling site Preloved (Paul *et al.* 2022). Popularity for brachycephalic breeds is driven by several factors, including behavioural characteristics and human lifestyle factors (Packer *et al.* 2019), but their distinctive appearance, particularly their ‘ *Kindchenschema* *’*-adhering flat faces (baby schema, whereby dogs retain infant features into adulthood [Lorenz 1971; Paul *et al.* 2023a,b]), has been reported as the predominant driver of their acquisition (Packer *et al.* 2019). Alternative breeds or crossbreeds with less extreme conformations that fulfil the desires of this ownership niche could offer major longer-term welfare gains, but evidence is currently lacking on which alternatives would be popular. Theoretical frameworks in behavioural science suggest that interventions that target behavioural skill, such as offering specific alternatives or replacement behaviours, are generally more effective than interventions that only target knowledge or attitudes (Albarracín *et al.* 2024). This implies that telling someone what not to do (i.e. ‘don’t buy a brachycephalic dog’), without suggesting what to do instead (e.g. suggesting buying another breed or crossbreed without extreme brachycephalic conformation), holds a lower probability of successful behaviour change. Evidence to generate welfare-focused recommendations on alternative breeds or crossbreeds for those seeking to acquire a brachycephalic dog are urgently needed.

Outcrossing (breeding between dogs of different breeds or between dogs of the same breed that share limited ancestry with the intention to retain the original breed status) or crossbreeding (intentional crossing between different breeds with the intention of creating new breeds, sometimes called designer breeds) between a brachycephalic breed with a non-brachycephalic breed could be theorised to result in more moderate features in the offspring and therefore a reduction in disease burden compared to the extreme parent breed. However, there are limited data available on the health of brachycephalic outcrosses, or brachycephalic ‘designer’ crossbreeds. ‘Retropugs’ (a designer crossbreed created in Germany by crossing purebred Pugs and Parson Russell Terriers) have been demonstrated to perform better than purebred Pugs in respiratory tests (Bartels *et al.* 2015). However, the small size of that study which included just seven Retropugs and 42 Pugs limits interpretation, and the study design provided data only on respiratory health. Further research is therefore urgently needed on the broader health, welfare and behaviour status of brachycephalic designer crossbreeds compared to their purebred progenitor breeds to determine whether they can be recommended as good alternatives to owners seeking to acquire a purebred extreme brachycephalic dog.

To start to fill these information gaps, the current study aimed to compare specific health problems, behaviours and dog-owner relationship between purebred Pugs and two popular Pug-crosses: Puggles (Pug × Beagle) and Jugs (Pug × Jack Russell Terrier). The current study hypothesised that each of the two Pug-crossbreeds (i.e. Jug, Puggle) would show reduced conformation-related disease burden across body systems, including BOAS, whilst retaining similar behavioural traits and dog-owner relationships.

## Materials and methods

The study received ethical approval from the Clinical Research Ethical Review Board at the Royal Veterinary College (URN 2023 2220-2). Owners of Pugs, Jugs and Puggles were recruited using direct and snowball sampling via digital channels between November 2023 and March 2024. Posters (Supplementary material S1) were placed on group pages on social media sites such as Facebook which included the words ‘Pug’ ‘Jug’ or ‘Puggle’ and were also shared by the project’s three funding bodies (Animal Welfare Foundation, RSPCA and Blue Cross) on their social media. Some UK veterinary practices engaged clients with the project via social media and physical posters in waiting rooms.

A QR code on the poster directed interested owners to a sign-up page to leave their name, contact email address, location (nearest town or postcode), whether they owned a Pug, Jug or Puggle, and the age of the dog. Study inclusion criteria consisted of: UK-based and available for assessment on dates specified, owned a Pug, Jug or Puggle between the age of 1–8 years (to reduce impact of age-related cognition change).

Based on previous evidence that 74.4% of Pugs score RFGS Grade 1, 2 or 3 (Liu *et al.* 2017), power calculations indicated a minimum sample size required for each breed group (Pug, Jug, Puggle) was n = 19 (α = 0.05, power = 0.80 and assuming that 25% or less of the Pug designer crossbreeds scored RFGS Grade 1, 2 or 3). To account for greater predicted phenotypic and other conformational health variability in the Pug-crossbreed groups compared to purebred Pugs, we aimed to recruit double this sample per Pug-crossbreed (n = 38), with the aim of a minimum total of 76 crossbreed dogs assessed.

Respondents to the sign-up page were sorted geographically by UK county. For logistical reasons, starting with the county with the most respondents, all applicants per county were contacted directly by EY to assess for study eligibility and to arrange a visit to assess their dog. Assessments ran from January to September 2024, when the target number of dogs from each of the three breeds had been met or exceeded. All assessments were performed by EY, a veterinary surgeon with 11 years of clinical experience in companion animal practice and trained in RFGS assessment.

### Owner questionnaire

During the face-to-face assessment, owners completed a questionnaire to collect background information on themselves and their dog, and objective and subjective reports of their dog’s health. The first section included basic owner demographic information: owner age, gender and whether they were the main carer for the dog. The second section included demographic information about the dog being assessed: breed (including generation number from first cross-breeding if a Jug or Puggle), Royal Kennel Club (RKC) registration status (Pugs only), colour, sex, neuter status and age at neutering, date of birth, age at acquisition, price paid for their dog, and details surrounding acquisition.

The next section focused on owner-reported information on the dog’s health, including reproductive history and veterinary diagnosed disorders. Owners reported their dog’s estimated total veterinary costs to date (in £), which were used to calculate veterinary costs per month of ownership based on age at acquisition and current age. Owner perceptions were collected for their dog’s veterinary costs, exercise levels, behaviour and maintenance that were reported as ‘less than expected’, ‘met expectations’ or ‘more than expected’. Owners reported all diagnoses by a veterinary surgeon in their dog of the following disorder types: breathing problems, eye problems, orthopaedic problems, skin disease, ear disease or dental disease. Owners also reported any previous surgical treatment for BOAS.

Owners rated their dog’s health compared to their expectations of the health of a purebred brachycephalic dog (e.g. Pug), a crossbreed brachycephalic dog (e.g. a Jug or Puggle) and a non-brachycephalic dog (e.g. Labrador Retriever) on an established five-point scale from ‘much less healthy than average for breed’ to ‘much healthier than average for breed’ (Packer *et al.* 2019). Owners were asked to report whether they considered their dog to have a ‘problem’ across four established dysfunction domains related to brachycephaly: ‘heat regulation’, ‘sleeping’, ‘breathing’, ‘eating’ and one novel domain ‘exercise/playing’ (Packer *et al.* 2019). Owners reported their perception of the severity of common brachycephaly-associated airway impairments in their dog on established scales across four areas: breathing sounds/breathing difficulty (the Owner Reported Breathing Score; ORB; [Packer *et al.* 2012]), heat intolerance, eating difficulties and sleep dysfunction (Packer *et al.* 2019). The scoring systems combined scores across a number of sub-questions on breathing sounds (four questions, scores: 0–20), breathing difficulty (four questions, scores: 0–20), heat intolerance (three questions, scores: 0–12), eating difficulties (four questions, scores: 0–20) and sleep dysfunction (six questions, scores: 0–30). See Supplementary material S2. The Canine Brief Pain Inventory (CBPI; ten questions, scores: 0–100) was used to assess owners’ perceptions of whether their dog had any chronic pain and their overall quality of life (Brown *et al.* 2007).

The owners used the shortened version (29 questions) of the validated canine behavioural assessment and research questionnaire (C-BARQ) to report their dog’s behaviour (Wilkins *et al.* 2024). This version of the C-BARQ includes six scales: excitability, aggression, fear and anxiety, separation anxiety, attachment and attention-seeking behaviour and trainability. Two question subsets of the Monash Dog Owner Relationship Scale (MDORS) (Dwyer *et al.* 2006) were used to assess the perceived emotional closeness (ten questions) and perceived ‘costs’ of dog ownership (i.e. beyond financial) (nine questions) for all owners. All questions were scored 1–5 by the owner (strongly disagree to strongly agree), with higher scores for emotional closeness representing high relationship quality, whereas increasing scores for the perceived costs subset represents lower relationship quality.

### Practical assessment

A range of morphological, clinical and cognitive metrics were assessed for all dogs, with all assessments performed by a single rater (EY).

#### Morphological assessments

Body condition score (BCS) was assessed using the WSAVA 9-point scale (WSAVA 2013). Morphological measurements were taken using established breed-defining measurement protocols (Sutter *et al.* 2008), including muzzle length, cranial length, head width, eye width, palpebral fissure width, neck length, neck girth, chest girth, chest width, body length, height at the withers and height at the base of tail. All measurements were made to the nearest millimetre and taken using a standard soft measuring tape. Muzzle length was defined as the distance from the dorsal tip of the nasal planum to the stop. Cranial length was defined as the distance from the stop to the occipital protuberance, following the curve of the cranial surface. The degree of brachycephaly (facial foreshortening) was quantified by the craniofacial ratio (CFR): the muzzle length divided by the cranial length (Packer *et al.* 2012). Palpebral fissure width was measured consciously using the unstretched-eyelid method with a soft tape measure pulled taut from the medial to lateral canthus (Packer *et al.* 2015a). To make palpebral fissure width relative to overall skull size, and therefore comparable between differently sized dogs, it was divided by cranial length, with relative palpebral fissure width = (palpebral fissure width [mm]/cranial length [mm] × 100) (Packer *et al.* 2015a). Nostril stenosis was graded on the four-point scale described in Liu *et al.* (2016). All dogs were examined for the presence of a nasal fold; defined as a discernible fold of skin on the dorsal surface of the muzzle, present without manipulation of the skin. This was recorded as a binary trait and also categorised as broken (i.e. segmented over the nasal bridge) or unbroken (i.e. runs continuously over the nasal bridge) and the depth (mm) of the fold measured with digital callipers. All dogs were also assessed for the presence of tail fold and skin folds elsewhere on the body. All dogs were examined unrestrained for scleral visibility with the dog looking directly forwards (Packer *et al.* 2015a). A scleral score was given from 0–4, based on whether sclera was visible (0 = not visible, 1 = visible) in four quadrants (medially, laterally, dorsally or ventrally). Tail length and shape (straight, curled, double-curled, screw tail) was recorded for all dogs, and all were assessed for whether the tail could be moved in a ventral-dorsal plane.

#### Clinical assessment

All dogs underwent formal RFGS assessment (Royal Kennel Club 2026) with a registered assessor (EY), who had undergone the requisite training via The Royal Kennel Club. The RFGS is based on clinical evaluation of respiratory signs before and immediately after a 3-min exercise tolerance test (Liu *et al.* 2015). Thoracic auscultation was performed using a Littmann Classic III stethoscope, with resting heart rate, resting respiratory rate and any abnormalities recorded. Each dog was graded using the established four-point functional grading system (where 0 = respiratory noise not present and no respiratory effort to 3 = severe respiratory noise with dyspnoea), with grades 0–1 considered clinically unaffected by BOAS in previous studies and grades 2–3 clinically affected (Ladlow 2021).

All dogs with skin folds were further assessed for the presence of skin fold dermatitis in all skin fold locations present (e.g. nasal fold, tail fold, or other skin folds). Both ears were assessed for erythema, stenosis and discharge, with each characteristic scored from 0–3 (where 0 = absent and 3 = severe), giving a potential total ear score from 0–18. All dogs were assessed for the presence of entropion, ectropion and prolapsed nictitating membrane gland (PNMG) in both eyes.

All dogs underwent a conscious dental health assessment based on protocols from the Veterinary Oral Health Council and on advice from the British Veterinary Dental Association. Halitosis, discoloured saliva, missing/mobile/fractured/discoloured teeth, retained deciduous teeth and dental crowding were recorded as binary traits. Calculus and gingivitis were scored from 0–3 and dental malocclusion was recorded as Class 1, 2, 3 or 4 according to the American Veterinary Dental College nomenclature (AVDC 2022).

A basic neurological assessment was adapted from Rylander (2013) and performed for all dogs, with abnormalities of mentation, posture, gait, postural reactions (paw placing, visual/tactile placing, hopping and wheelbarrow), palpation of spine and neck, flexibility and patella luxation assessed.

#### Cognitive assessment

All dogs participated in a food-searching cognitive test (González-Martínez 2013). The dog was gently restrained by the owner whilst the dog was shown a food reward (1 cm^3^ piece of freshly boiled chicken). The treat was then placed under a 20 × 10 cm (length × width) clear plastic container, positioned 100 cm from the dog. The dog was released as a timer started. The task was scored as: (1) obtains food within 2 min (latency to obtain food reward recorded); (2) attempts to get food reward but does not obtain it within 2 min; (3) sniffs container but does not attempt to get food reward; and (4) makes no attempt to get food reward.

### Statistical analysis

Following initial cleaning of data in Microsoft Excel®, statistical analyses were carried out in IBM SPSS® Statistics (V29.0.0.0). Initial data analysis included calculation of descriptive statistics for all variables: frequency and percentage for categorical variables, mean and standard deviation (SD) for normally distributed continuous variables, and range, median and interquartile range for non-normally distributed continuous variables. Normality of data distribution was ascertained by visual inspection of histograms. Data from Pugs, Jugs and Puggles were compared at the univariable level using chi-squared (*X*^2^) analysis for categorical variables, ANOVA for normally distributed continuous data and Kruskal-Wallis tests for non-normally distributed continuous data.

Using R version 4.3.1 (R Core Team 2025) with the logistf and dplyr packages, a multivariable logistic regression model was used to analyse the association between predictors including breed, age, sex, BCS, nostril grade, and CFR, and an outcome of RFGS grade (binary outcome: negative (grade 0/1) / positive (grade 2/3). Initial inspection revealed a strong correlation between breed and CFR, and preliminary models including both variables resulted in unstable coefficient estimates and quasi-complete separation. To address this, Firth’s penalised likelihood logistic regression was performed using the logistf package in R. The final multivariable model was created using an information theory approach to include all predictor variables of interest regardless of their statistical significance, and included breed, age, sex, BCS, and nostril grade as predictors. We report coefficient estimates, odds ratios (ORs), 95% confidence intervals (CIs), and *P*-values. Model fit was assessed using the Hosmer-Lemeshow test. Statistical significance was set at the 5% level.

## Results

The study included assessments of 150 dogs: Pugs (34%; n = 51); Jugs (34%; n = 51); and Puggles (n = 32%; n = 48).

### Owner demographics

Owners (n = 150) were predominantly female (n = 130; 86.7%), with no statistically significant difference in gender distribution between Pug, Jug and Puggle owners (female, Pug: 90.2% vs Jug: 84.3% vs Puggle: 85.4%; *X*^2^ = 0.859; *P* = 0.651). The most common age group was 36–45 years old (Pug: 37.5% vs Jug: 32.5% vs Puggle: 30.0%) followed by 46–55 years old (Pug: 43.6% vs Jug: 33.3% vs Puggle: 23.1%) with no difference in owner age groups between breeds (*X*^2^ = 10.84; *P* = 0.370).

### Dog demographics

Both Pug crossbreeds were predominantly first generation (Jug: 60.8% vs Puggle: 56.3%) with no significant difference in generation between Jug and Puggle (*X*^2^ = 0.238; *P* = 0.888). Of the purebred Pugs, n = 18 (35.3%) were registered with the Royal Kennel Club. The median age of the dogs at time of assessment was 48 months (IQR: 30–72, range: 15–100) with no significant difference between the three breeds (*X*^2^ = 12.200; *P* = 0.590). The majority of dogs were neutered (Pug: n = 40 [78.5%] vs Jug: n = 44 [86.3%] vs Puggle: n = 36 [75%]) which significantly differed between breeds (*X*^2^ = 12.941; *P* = 0.044). A greater proportion of Pugs had birthed or sired a litter compared to the crossbreed dogs (Pug: n = 11 [21.6%] vs Jug: n = 4 [7.8%] vs Puggle: n = 2 [4.2%]; *X*^2^ = 10.761; *P* = 0.029).

### Acquisition factors

The median age at acquisition was 2.5 months (IQR: 2–8, range: 0–54) with no significant difference between breeds (*X*^2^ = 44.397; *P* = 0.109). The median purchase price was significantly higher for Pugs at £1,075 (IQR: £700–£1,250, range: £0–£2,200), compared to Jugs (£500, IQR: £200–£800, range £0–£2,000) and Puggles (£600, IQR: £400–£825, range: £0–£1,700) (KW: 10.95; *P* = 0.004).

### Owner-reported health, healthcare costs and owner expectations

Most participating dogs were insured (Pug: n = 32 [62.7%] vs Jug: n = 37 [72.5%] vs Puggle: n = 33 [68.8%]) with no significant difference between breeds (*X*^2^ = 9.716; *P* = 0.285). Median estimated veterinary costs per month of current ownership were significantly higher for Pugs than for either Jugs or Puggles (Pug: £41.67, IQR: £11.23–£92.41 vs Jug: £9.65, IQR: £6.12–£13.34; vs Puggle: £10.17, IQR: £5.83–£15.12; KW: 46.03, df = 2; *P* < 0.001).

Expectations on veterinary costs varied statistically significantly between the breeds (*P* = 0.008), with Pug owners least likely to consider perceiving costs as lower than expected (Table 1). Jug and Puggle owners were significantly less likely to rate their dogs’ exercise requirements as lower than expected compared to Pug owners. Pug owners were more likely to rate their dog’s behaviour as better than expected compared to Jug and Puggle owners, while Puggle owners were more likely to rate their dog’s behaviour as worse than expected compared to Jug and Pug owners. There was no significant difference between breeds for maintenance expectations (Table 1).

**Table 1.** Frequency and percentage of Pug, Jug and Puggle owners’ expectations of ownership regarding veterinary costs, exercise needs, behaviour and maintenance needs. Figures highlighted in **bold** indicate statistically significant results. Table 1. long description.

Domain	Rating	Pug (n = 51)	Jug (n = 51)	Puggle (n = 48)	*X*^2^	*P*-value
Veterinary costs	Below expectations	5 (9.8%)	12 (23.5%)	14 (29.8%)	13.681	**0.008**
	Met expectations	32 (62.7%)	34 (66.7%)	30 (62.5%)		
	Above expectations	14 (27.5%)	5 (9.8%)	3 (6.4%)		
Exercise	Below expectations	9 (17.6%)	0 (0.0%)	0 (0.0%)	20.197	**< 0.001**
	Met expectations	30 (58.8%)	33 (64.7%)	36 (76.6%)		
	Above expectations	12 (23.5%)	18 (35.3%)	11 (23.4%)		
Behaviour	Below expectations	4 (7.8%)	4 (7.8%)	7 (14.9%)	10.098	**0.039**
	Met expectations	36 (70.6%)	45 (88.2%)	36 (76.6%)		
	Above expectations	11 (21.6%)	2 (3.9%)	4 (8.5%)		
Maintenance	Below expectations	3 (5.9%)	1 (2.0%)	2 (4.3%)	9.361	0.053
	Met expectations	40 (78.4%)	49 (96.1%)	43 (91.5%)		
	Above expectations	8 (15.7%)	1 (2.0%)	2 (4.3%)		

From owner reports, Pugs were significantly more likely to have been diagnosed with respiratory and ocular health problems compared to Puggles and Jugs (Table 2). The most common disorder type overall was dental (around 1 in 5 of all dogs across breeds), which did not differ significantly between breeds (*P* = 0.937).

**Table 2.** Frequency and percentage of owner-reported health problems in Pugs, Jugs and Puggles. Figures highlighted in **bold** indicate statistically significant results Table 2. long description.

Reported health problem	Pug (n = 51)	Jug (n = 51)	Puggle (n = 48)	*X*^2^	*P*-value
Dental	11 (21.6%)	10 (19.6%)	9 (18.8%)	0.130	0.937
Orthopaedic	3 (5.9%)	3 (5.9%)	6 (12.5%)	1.942	0.379
Ocular	18 (35.3%)	3 (5.9%)	6 (12.5%)	17.466	**< 0.001**
Skin	5 (9.8%)	1 (2.0%)	4 (8.3%)	2.836	0.242
Ears	3 (5.9%)	5 (9.8%)	5 (10.4%)	0.767	0.681
Respiratory	11 (21.6%)	0 (0.0%)	0 (0.0%)	23.043	**< 0.001**

Pug owners were most likely to perceive that their dog had a problem with heat (Pug: n = 22 [43.1%] vs Jug: n = 3 [6.0%] vs Puggle: n = 6 [12.5%]; *X*^2^ = 24.098; *P* < 0.001), breathing (Pug: n = 15 [29.4%] vs Jug: n = 0 [0.0%] vs Puggle: n = 2 [4.2%]; *X*^2^ = 25.283; *P* < 0.001) and exercise (Pug: n = 9 [17.6%] vs Jug: n = 2 [4.0%] vs Puggle: n = 1 [2.1%]; *X*^2^ = 9.759; *P* = 0.008) compared to Jug and Puggle owners. No significant difference was detected between breeds in owner-reported sleep or eating problems.

Pugs scored significantly higher (i.e. worse) in owner-reported dysfunction scores for heat, eating, sleep, and breathing compared to Jugs and Puggles (Table 3). For owner-reported breathing (ORB) dysfunction scores, 51.0% (n = 26) Pugs received a score of 8 or above, indicating clinically relevant airway disease (Packer *et al.* 2012), compared to 2.0% (n = 1) of Jugs and 4.2% (n = 2) of Puggles.

**Table 3.** Median and interquartile ranges for owner-reported dysfunction scores for Pugs, Jugs and Puggles across heat, eating, sleeping and breathing domains. Figures highlighted in **bold** indicate statistically significant results Table 3. long description.

Owner reported dysfunction score	Pug (n = 51)	Jug (n = 51)	Puggle (n = 48)	Kruskal-Wallis	*P*-value
Heat (scored out of 12)	4.0 (0–8)	2.0 (0–6)	2.0 (0–8)	24.42	**< 0.001**
Eating (scored out of 20)	3.0 (0–11)	0.0 (0–6)	1.0 (0–5)	19.54	**< 0.001**
Sleeping (scored out of 30)	6.0 (0–18)	2.0 (0–11)	1.0 (0–7)	29.23	**< 0.001**
Breathing (ORB) (scored out of 40)	8.0 (0–22)	2.0 (0–10)	2.0 (0–10)	42.22	**< 0.001**

Mean (± SD) Canine Brief Pain Inventory scores were significantly higher for Pugs (12.53 [± 5.53], 95% confidence interval [CI]: 10.97–14.08) than for Jugs (10.35 [± 1.99], CI: 9.89–11.01) and Puggles (10.21 [± 1.44], CI: 9.79–10.63) (*F*-statistic = 6.60; *P* = 0.002).

**Figure 1.**
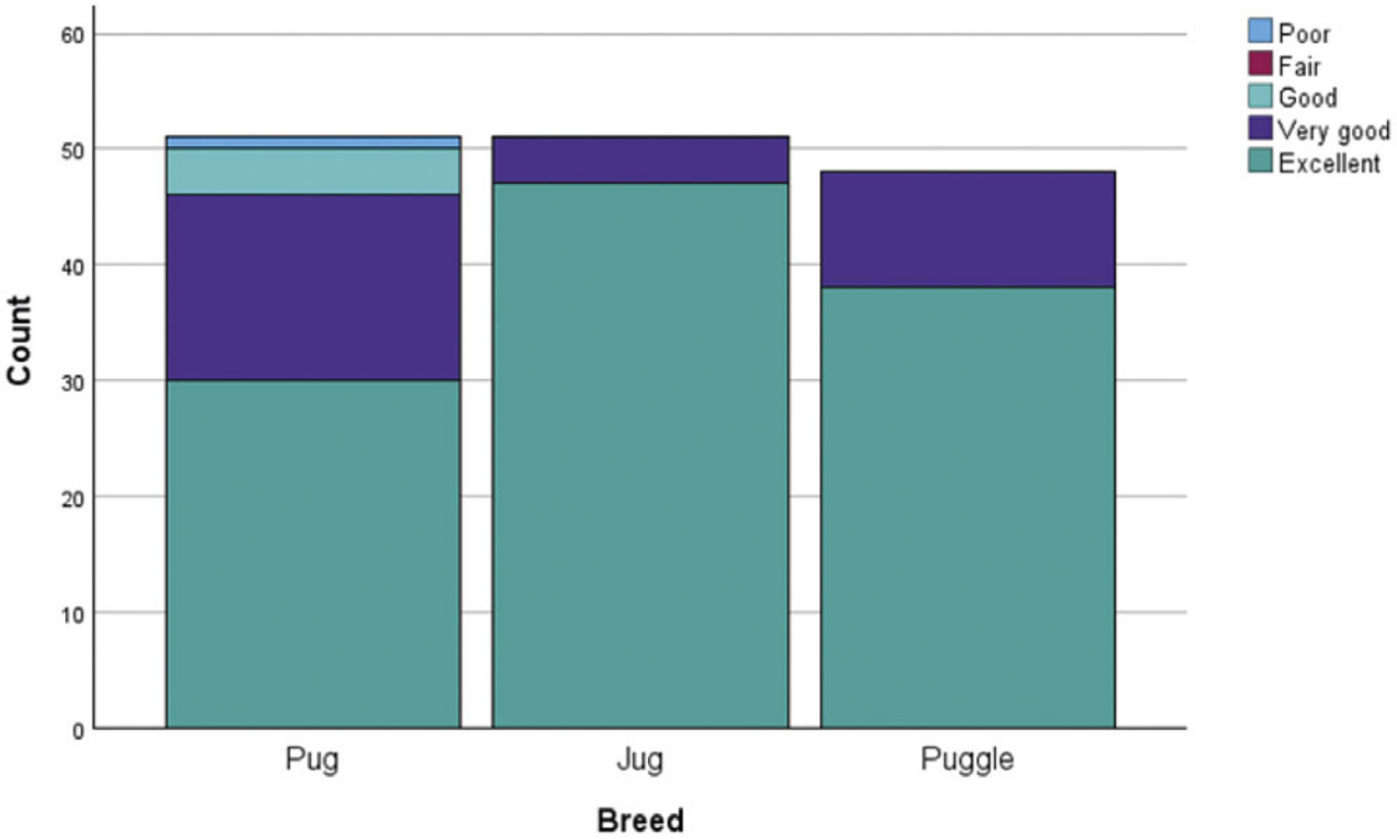
Owner-reported quality of life scores for Pugs (n = 51), Jugs (n = 51) and Puggles (n = 48).

There was a statistically significant difference between breed groups when owners rated their dog’s quality of life (*P* = 0.002), with Jug and Puggle owners more likely to rate their dog’s quality of life as ‘excellent’ compared to Pug owners (Pug: n = 30 [58.8%] vs Jug: n = 47 [92.2%] vs Puggle: n = 39 [81.2%]; *X*^2^ = 20.542) (Figure 1).

### Owner reported behaviour and attachment

Pugs and Jugs scored significantly higher in the owner-attachment and attention-seeking scale of the C-BARQ (i.e. more attached and more attention-seeking behaviour) compared with Puggles, while Pugs scored significantly lower in the trainability scale (i.e. harder to train) compared to Jugs and Puggles (Table 4). No statistically significant differences were detected between breeds for the remaining four scales (excitability, aggression, fear and anxiety and separation anxiety) (Table 4).

**Table 4.** Owner-reported C-BARQ scores for Pugs, Jugs and Puggles. Figures highlighted in **bold** indicate statistically significant results. Table 4. long description.

C-BARQ scale	Pug median score (n = 51)	Jug median score (n = 51)	Puggle median score (n = 48)	Kruskal-Wallis	*P*-value
Excitability	7.00	7.00	6.00	2.715	0.257
Aggression	5.00	6.00	8.00	5.340	0.070
Fear and anxiety	8.00	10.00	8.00	0.084	0.959
Separation	2.00	2.00	1.00	3.149	0.207
Attachment and attention-seeking	7.00	7.00	6.00	8.890	**0.012**
Trainability	7.00	8.00	8.00	9.929	**0.007**

Median MDORS scores did not differ significantly between breeds for either the perceived emotional closeness subscale (Pug: 45, IQR 25–50 vs Jugs: 47, IQR 40–50 vs Puggles: 45, IQR 33–50; KW 6.018; *P* = 0.056) or perceived costs subscale (Pug: 10, IQR 9–16 vs Jugs: 11, IQR 9–18 vs Puggles: 12, IQR 9–21; KW 6.017; *P* = 0.057).

### Physical assessment

#### Morphometrics

**Figure 2.**
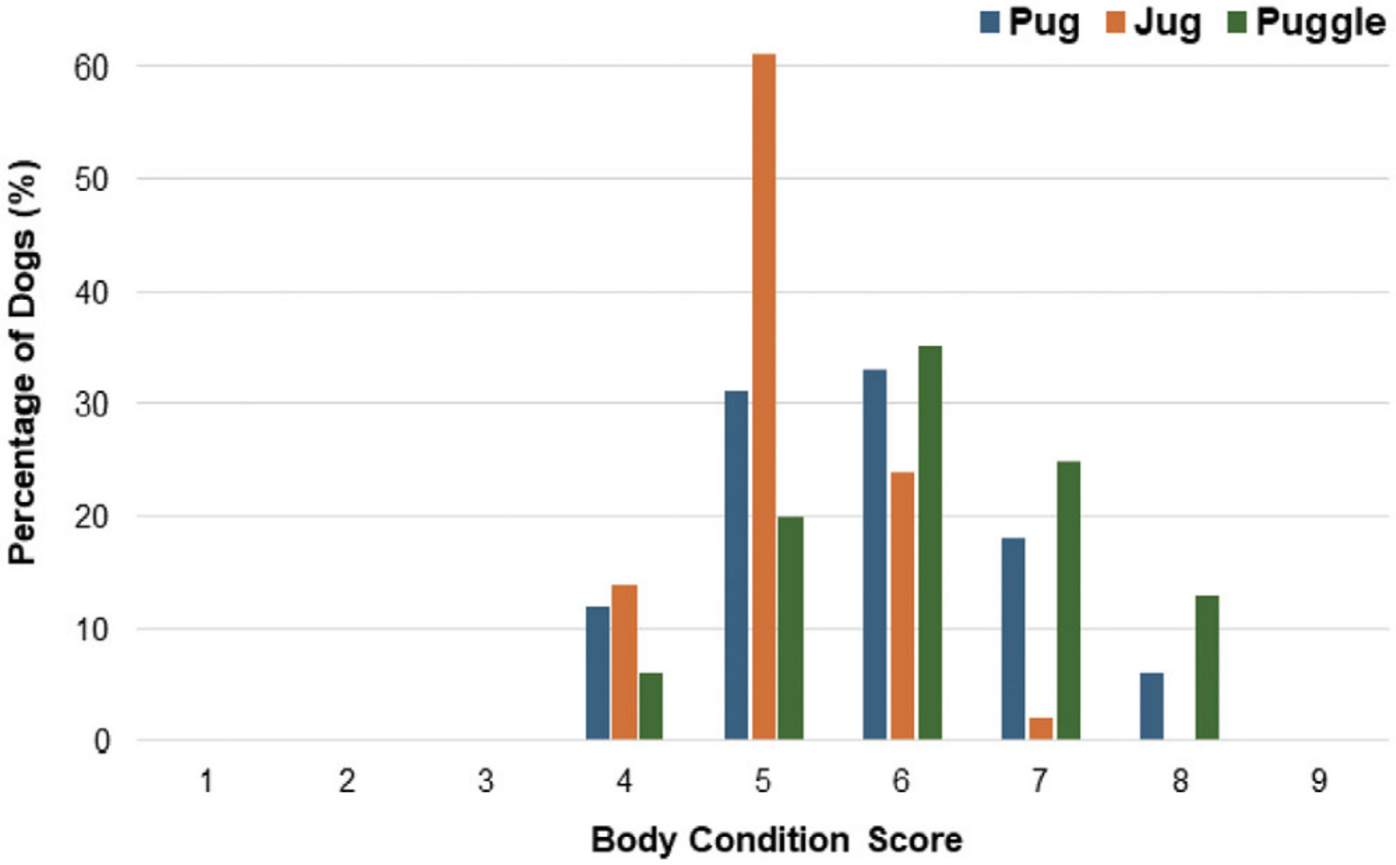
Body condition scores (BCS) for Pugs (n = 51), Jugs (n = 51) and Puggles (n = 48).

The median BCS for all study dogs was 6/9 (IQR 5–6, range 4–8) but varied significantly between breeds (*X*^2^ = 29.839; *P* < 0.001) (Figure 2). Three-quarters of Jugs (n = 38; 74.5%) showed an ideal BCS of 4–5, compared to under half of Pugs (n = 22; 43.1%) and only around one-quarter of Puggles (n = 13; 27.1%). In comparison, over one-third of Puggles (n = 18; 37.5%) and around one-quarter of Pugs (n = 12; 23.5%) were severely overweight with a body condition of 7–8, compared to just one Jug (1.9%).

**Figure 3.**
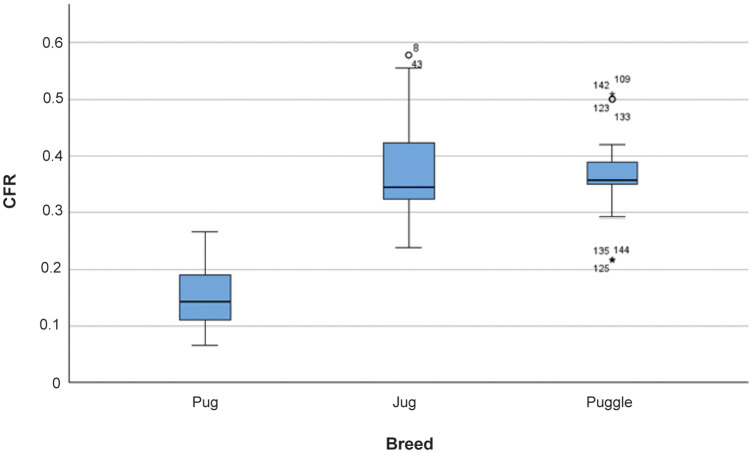
Craniofacial ratio (CFR) scores for Pugs (n = 51), Jugs (n = 51) and Puggles (n = 48).

**Figure 4.**
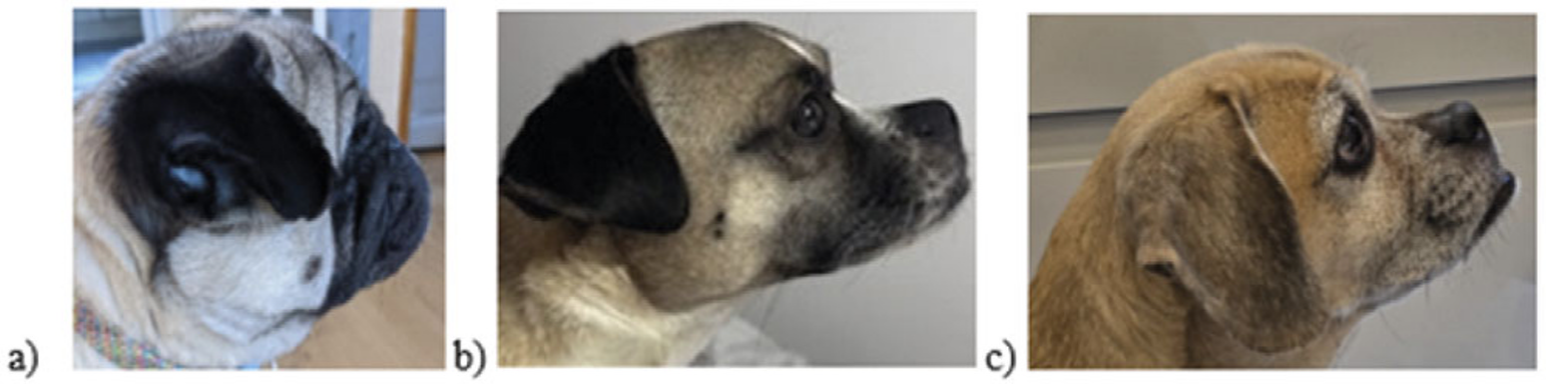
Study photos of an (a) Pug, (b) Jug and (c) Puggle demonstrating differences in craniofacial ratio (CFR) between breeds.

Pugs exhibited a significantly lower CFR (0.15 [± 0.05]) compared to Jugs (0.38 [± 0.09]) and Puggles (0.36 [± 0.06]) (Figures 3 and 4).

Around half of Jugs (n = 25; 49.0%) and Puggles (n = 24; 50.0%) scored a 0 scleral score, compared to only 4.0% (n = 2) of Pugs. Conversely, 22.0% (n = 11) of Pugs had a scleral visibility score of 3 or 4, compared to none of the Jugs or Puggles, which significantly differed between breeds (*X*^2^ = 55.043; *P* < 0.001).

Puggles were overall larger than Pugs and Jugs and had a significantly larger head width, neck length, neck girth, chest girth, body length and body height measurement in comparison to Pugs and Jugs (Table 5).

**Table 5.** Body conformational measurements of Pugs (n = 51), Jugs (n = 51) and Puggles (n = 48). Figures highlighted in **bold** indicate statistically significant results Table 5. long description.

Body measurement	Pug mean (± SD) value (n = 51)	Jug mean (± SD) value (n = 51)	Puggle mean (± SD) value (n = 48)	F-statistic	*P*-value
Muzzle length (mm)	15.53 (± 4.94)	39.39 (± 7.55)	40.06 (± 7.69)	211.768	**< 0.001**
Cranial length (mm)	104.20 (± 13.36)	105.59 (± 11.44)	111.65 (± 14.64)	4.426	**0.014**
Craniofacial ratio (CFR)	0.15 (± 0.05)	0.38 (± 0.09)	0.36 (± 0.06)	161.527	**< 0.001**
Eye width (mm)	55.88 (± 8.32)	42.63 (± 7.36)	46.31 (± 7.29)	40.406	**< 0.001**
Palpebral fissure (mm)	26.32 (± 4.50)	28.65 (± 4.00)	34.23 (± 5.03)	39.454	**< 0.001**
Relative palpebral fissure width	25.25 (± 3.38)	27.39 (± 3.54)	30.84 (± 3.98)	10.921	**0.006**
Head width (mm)	123.76 (± 11.61)	121.18 (± 13.48)	137.92 (± 23.34)	14.12	**< 0.001**
Neck length (mm)	151.71 (± 21.58)	151.27 (± 23.08)	186.21 (± 30.20)	31.178	**< 0.001**
Neck girth (mm)	359.00 (± 30.79)	316.47 (± 25.75)	368.96 (± 49.54)	29.24	**< 0.001**
Chest girth (mm)	522.31 (± 52.06)	496.78 (± 40.92)	610.71 (± 90.87)	42.438	**< 0.001**
Body length (mm)	351.27 (± 37.07)	337.53 (± 22.89)	410.46 (± 52.07)	48.673	**< 0.001**
Height at withers (mm)	320.88 (± 28.10)	340.29 (± 38.48)	372.83 (± 48.07)	22.457	**< 0.001**
Height at tail-base (mm)	313.08 (± 29.23)	335.69 (± 5.45)	374.17 (± 47.28)	30.908	**< 0.001**

**Figure 5.**
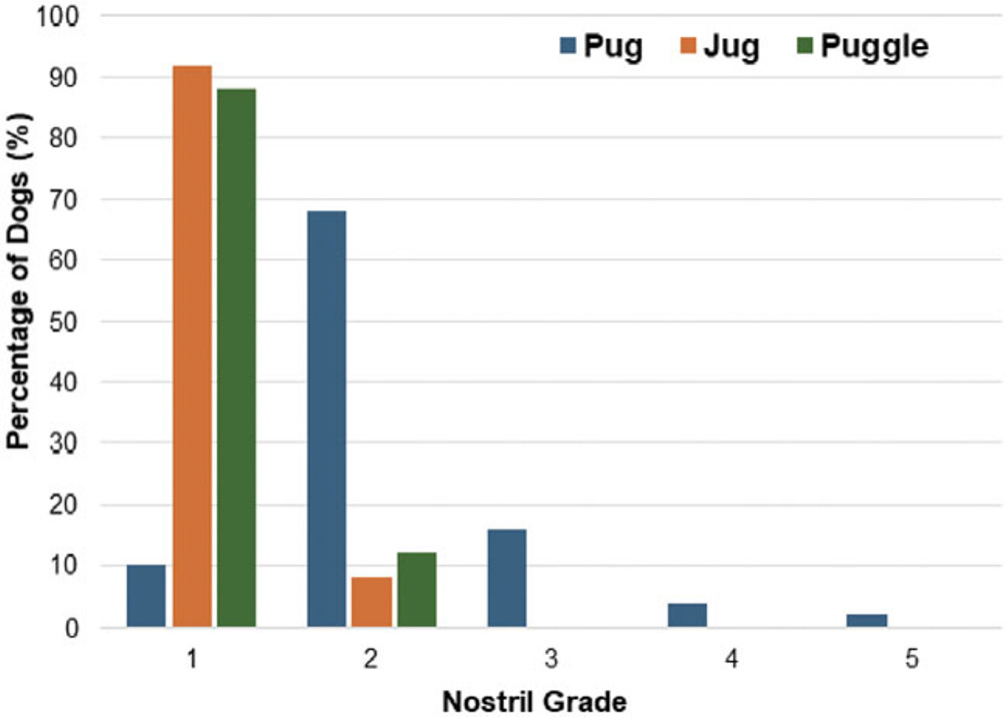
Nostril grade scores for Pugs (n = 51), Jugs (n = 51) and Puggles (n = 48). Higher scores represent more severe stenosis.

Nostril grades significantly differed between breeds (*X*^2^ = 94.508; *P* < 0.001). The majority of Jugs (n = 47; 92.2%) and Puggles (n = 42; 87.5%) had a Grade 1 nostril score, compared to only n = 5 (9.8%) of Pugs. The majority (n = 35; 68.6%) of Pugs had a Grade 2 nostril score, compared to n = 4 (7.8%) of Jugs and n = 6 (12.5%) of Puggles. Grade 3–5 nostril scores were only seen in Pugs, where one in five Pugs (n = 11; 21.6%) were affected (Pugs: grade 3: n = 8 [15.7%]; grade 4: n = 2 [3.9%]; grade 5: n = 1 [2.0%]) (Figure 5).

### Clinical assessments

**Figure 6.**
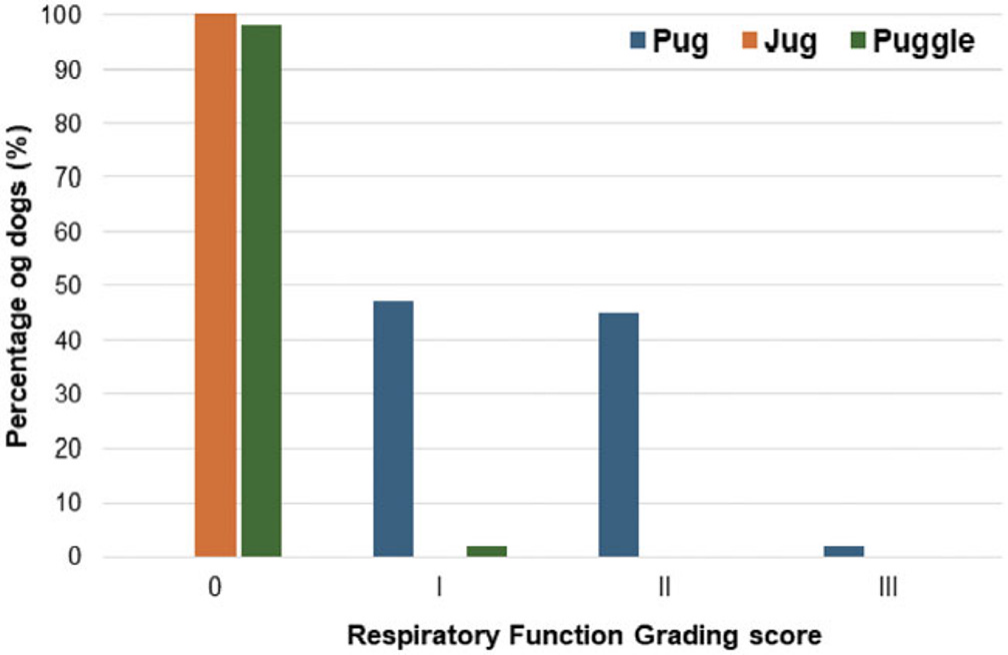
Respiratory Function Grading (0–3) for Pugs (n = 51), Jugs (n = 51) and Puggles (n = 48), where a higher score represents more severe respiratory dysfunction due to Brachycephalic Obstructive Airway Syndrome (BOAS).

The RFGS results significantly differed between the three breeds (*X*^2^ = 145.803; *P* < 0.001) (Figure 6). Three (5.9%) Pugs had a history of corrective surgery for BOAS and were excluded from analysis. No Jugs or Puggles had a history of corrective surgery. No Pugs were graded RFGS Grade 0 (indicating normal respiratory function), whereas all Jugs (n = 51; 100%) and the majority of Puggles (n = 47; 97.9%) were graded 0. The majority of Pugs were graded RFGS Grade 1 (n = 24; 47.1%) or Grade 2 (n = 23; 45.1%). Among the Puggles, only one dog (2.1%) was graded RFGS Grade 1, with the remainder being graded RFGS Grade 0.

CFR and breed were separately explored as variables of primary interest for association with RFGS grade as a binary outcome (grade 0/1 vs grade 2/3) through binary logistic regression modelling. Both models also considered age, sex, BCS and nostril grade as variables of secondary interest (Table 6). The CFR model included CFR as a continuous predictor. CFR was strongly and significantly associated with RFGS score. Higher CFR values were associated with substantially reduced odds of a positive RFGS grade (grades 2/3) (*P* = 0.018).

**Table 6.** Logistic regression results for RFGS grade (binary outcome: grade 0/1 vs grade 2/3) reporting odds ratio (95% CI) and *P*-values for models including either CFR or breed as the variable of primary interest. Figures highlighted in **bold** indicate statistically significant results. Table 6. long description.

Predictor	Category	Model 1 (CFR)			Model 2 (Breed)		
		OR (CFR)	95% CI (CFR)	*P*-value (CFR)	OR (Breed)	95% CI (Breed)	*P*-value (Breed)
(Intercept)		0.00	0.00–0.00	0.995	0.001	0.00–0.01	0.00
CFR	Continuous		0.00–0.02	**0.018**	–	–	–
Breed	Jug	Base	–	–	Base	–	–
	Pug	–	–	–	38.5	3.66–534.44	**< 0.001**
	Puggle	–	–	–	0.57	0.03–11.01	0.786
Age (months)	Continuous	1.07	0.70–1.65	0.745	1.11	0.81–1.55	0.518
Sex	Female entire	Base	–	–	Base	–	–
	Female neutered	2.94	0.45–19.40	0.341	1.64	0.25–12.81	0.609
	Male entire	29.00	0.48–1700	0.072	6.94	0.56–151.12	0.133
	Male neutered	5.60	0.53–58.72	0.155	2.37	0.35–19.67	0.379
BCS	Continuous	2.29	1.06–4.95	**0.035**	1.76	0.97–3.48	0.061
Nostril grade	Continuous	1.97	0.49–7.09	0.400	1.71	0.55–5.88	0.356

In the breed model, Pugs had markedly higher odds of a positive RFGS grade (grade 2/3) compared with Jugs (*P* < 0.001), while Puggles were not significantly different from Jugs (*P* = 0.786). Both the CFR and breed models showed similar effects of BCS, with an increase in BCS associated with higher odds of an RFGS positive grade (grade 2/3); however, this was a non-significant trend in the breed model. Age, sex, and nostril grade were not significantly associated with RFGS grade.

All (n = 51; 100%) Pugs had a nasal fold, compared to 15.7% (n = 8) of Jugs and 27.1% (n = 13) of Puggles. Pugs had a significantly higher prevalence of nasal fold dermatitis than Jugs or Puggles. Pugs were also significantly more likely to have skin folds on other parts of their body apart from their nose or tail than Jugs or Puggles (Table 7).

**Table 7.** Proportion of Pugs, Jugs and Puggles with skin folds and skin fold dermatitis at various locations across their body. Figures highlighted in **bold** indicate statistically significant results. Table 7. long description.

Skin fold location	Pug (n = 51)	Jug (n = 51)	Puggle (n = 48)	*X*^2^	*P*-value
Nasal fold	51 (100.0%)	8 (15.7%)	13 (27.1%)	84.999	**< 0.001**
Nasal fold dermatitis	18 (38.3%)	1 (2.0%)	0 (0.0%)	8.026	**0.018**
Tail fold	10 (19.6%)	6 (11.8%)	3 (6.3%)	4.045	0.132
Tail fold dermatitis	4 (7.8%)	2 (3.9%)	1 (2.1%)	0.090	0.956
Other skin folds	22 (43.1%)	5 (9.8%)	4 (8.3%)	23.830	**< 0.001**
Other skin fold dermatitis	6 (11.8%)	0 (0.0%)	1 (2.1%)	1.968	0.374

From a maximum score of 18 for ear pathology, around half of Pugs (n = 28; 54.9%) scored 0, which was significantly fewer (*P* < 0.001) compared to Puggles (n = 34; 66.7%) and Jugs (n = 41; 80.4%). One-fifth of Pugs (n = 10; 20.1%) had entropion, which was significantly higher than for Jugs (n = 2; 3.9%) or Puggles (n = 1; 2.1%; *X*^2^ = 12.118; *P* = 0.002). Ectropion was found in n = 3 (6.0%) of Pugs compared to none of the Jugs or Puggles (*X*^2^ = 6.062; *P* = 0.048). No significant difference was detected between the three breeds for PNMG (Pug: n = 3; 6.0% vs Jug: n = 2; 3.9% vs Puggle: n = 4; 8.3%; *X*^2^ = 0.848; *P* = 0.654). Jugs had significantly lower levels of halitosis and calculus compared to Pugs and Puggles. Grade 1 malocclusions were most commonly observed in Pugs, but Grade 3 malocclusions were most commonly observed in Puggles. Crowding and missing/loose teeth were significantly more common in Pugs and Jugs compared to Puggles (Table 8).

**Table 8.** Frequency and percentage of dental abnormalities seen in Pugs, Jugs and Puggles. Figures highlighted in **bold** indicate statistically significant results. Table 8. long description.

Dental abnormality		Pug (%) (n = 51)	Jug (%) (n = 51)	Puggle (%) (n = 48)	*X*^2^	*P*-value
Halitosis		21 (41.2%)	9 (17.6%)	17 (35.4%)	7.109	**0.029**
Calculus	Grade 0	14 (27.4%)	25 (49.0%)	17 (35.4%)	5.662	0.226
	Grade 1	26 (51.0%)	19 (37.3%)	24 (50.0%)		
	Grade 2	11 (21.6%)	7 (13.7%)	7 (14.6%)		
Gingivitis	Grade 0	8 (15.7%)	24 (47.1%)	21 (43.8%)	18.270	**0.006**
	Grade 1	34 (66.7%)	19 (37.3%)	16 (33.3%)		
	Grade 2	9 (17.6%)	8 (15.7%)	10 (20.8%)		
	Grade 3	0 (0.0%)	0 (0.0%)	1 (2.1%)		
Missing / loose teeth		26 (51.0%)	28 (54.9%)	15 (31.3%)	6.340	**0.042**
Retained deciduous teeth		10 (19.6%)	7 (13.7%)	3 (6.3%)	3.828	0.147
Dental crowding		37 (72.5%)	40 (78.4%)	27 (56.3%)	6.098	**0.047**
Malocclusion	Grade 0	9 (17.6%)	10 (19.6%)	13 (27.1%)	25.095	**< 0.001**
	Grade 1	16 (31.4%)	2 (3.9%)	2 (4.2%)		
	Grade 2	0 (0.0%)	0 (0.0%)	0 (0.0%)		
	Grade 3	25 (49.0%)	39 (76.5%)	33 (68.8%)		
	Grade 4	1 (2.0%)	0 (0.0%)	0 (0.0%)		

Pugs were significantly more likely to show reduced or absent response for: paw placing, visual/tactile placing, hopping and wheelbarrow responses compared to Jugs and Puggles. Pugs were significantly more likely to have reduced muscle and flexibility compared to Jugs and Puggles. Pugs were significantly more likely to have patella luxation compared to Jugs and Puggles (Table 9).

**Table 9.** Frequency and percentage of orthopaedic and neurological abnormalities seen in Pugs, Jugs and Puggles. Figures highlighted in **bold** indicate statistically significant results. Table 9. long description.

Orthopaedic abnormality	Pug (%) (n = 51)	Jug (%) (n = 51)	Puggle (%) (n = 48)	*X*^2^	*P*-value
Abnormal posture	1 (2.0%)	0 (0.0%)	0 (0.0%)	1.954	0.376
Gait: lame	5 (9.8%)	3 (5.9%)	5 (10.4%)	0.769	0.681
Gait: ataxic	2 (3.9%)	0 (0.0%)	2 (4.2%)	2.123	0.346
Gait: scuffing	3 (5.9%)	0 (0.0%)	0 (0.0%)	5.942	0.051
Paw placing	7 (13.7%)	0 (0.0%)	2 (4.2%)	8.851	**0.012**
Visual / tactile placing	6 (11.8%)	0 (0.0%)	1 (2.1%)	8.965	**0.011**
Hopping test	10 (19.6%)	1 (2.0%)	2 (4.2%)	11.838	**0.003**
Wheelbarrow test	7 (13.7%)	0 (0.0%)	2 (4.2%)	10.241	**0.006**
Reduced muscle	14 (27.5%)	3 (5.9%)	2 (4.2%)	17.807	**< 0.001**
Reduced flexibility	22 (43.1%)	6 (11.8%)	13 (27.1%)	12.638	**0.002**
Patella luxation	9 (17.6%)	3 (5.9%)	2 (4.2%)	6.398	**0.041**

The proportion of dogs passing the cognitive test within two minutes did not differ significantly between breeds (Pugs: n = 26; 51.0% vs Jug: n = 31; 60.7% vs Puggle: n = 30; 62.5%; *X*^2^ = 11.223; *P* = 0.082).

## Discussion

This is the first study to date to directly compare the health, behaviour and dog-ownership relationship of purebred Pugs with those of designer crossbreeds with a Pug progenitor (i.e. Jugs and Puggles). The findings reveal how crossbreeding between a breed with an extreme brachycephalic conformation (i.e. Pug) and a breed with more natural mesocephalic conformation (i.e. Jack Russell Terrier or Beagle) creates a new designer crossbreed that is phenotypically less extreme than the extreme progenitor breed and correspondingly has a much lower conformation-related disorder burden. These changes away from the extreme phenotype of the purebred Pug were achieved while still preserving similar owner-dog relationships and behavioural characteristics, which are traits valued by owners of extreme brachycephalic breeds (Packer *et al.* 2020, 2024). Given recent evidence that the UK public shows a consistent preference for less extreme brachycephalic dogs compared to the conformational level that is currently typical, the more moderate conformation of Pug crosses could appeal to many potential puppy buyers (Youens *et al.* 2025). Potential negative changes to behavioural characteristics have previously been highlighted as a concern for owners regarding crossbreeding extreme brachycephalics (Youens *et al.* 2026); however, these concerns were not supported by the current dataset, in which behavioural improvements were instead observed. The speed of improvement in health status achieved by crossbreeding is demonstrated by the majority of designer crossbreeds in this sample being first generation F1 crosses. This suggests that judicious crossbreeding can offer a more rapid and effective approach than the current slow or non-existent pace of conformational change resulting from restriction to within-breed selection strategies, that are often aimed at maintaining ‘purity’ of gene pools and adhering to current breed standards whilst attempting incremental improvements in breed health. It should be noted however that the predominance of F1 individuals in the current study sample may have influenced the apparent stability of the improvements observed. While first-generation crosses often show consistent phenotypic gains, greater variability may emerge in subsequent generations or with backcrossing, potentially reducing the uniformity or magnitude of these improvements. Accordingly, longitudinal monitoring of both conformational traits and associated health outcomes will be critical to ensure that gains are maintained over time.

### Physical assessment

The current study reveals a stark divide between Pugs and the two Pug-crosses regarding respiratory function. None (0/51; 0%) of the purebred Pugs scored a 0 (clinically unaffected) on the RFGS, compared to almost all (98/99; 99%) of Pug-crosses (Jugs and Puggles). These findings align with previous findings from a smaller study demonstrating less impaired respiratory function in Pug-crosses with less extreme conformation compared to purebred Pugs (Bartels *et al.* 2015). The results from the current study highlight the current crisis of respiratory health in purebred Pugs, with almost half assessed as suffering from clinical BOAS to the level of requiring veterinary intervention, scoring a Grade 2 (moderate) or Grade 3 (severe) (Royal Kennel Club 2026).

Previous studies have identified that the most extreme brachycephalic conformations have the highest risk for BOAS, with risk rising as relative muzzle length shortens (Packer *et al.* 2015b; Tomlinson *et al.* 2025, 2026). In the current study, we show that the two Pug crosses – Jugs and Puggles – had a significantly increased muzzle length and craniofacial ratio compared to purebred Pugs, and that this higher CFR predicted a lower risk of BOAS as assessed by the RFGS. Jugs and Puggles had a substantially highly decreased risk of BOAS compared to Pugs, as shown by their RFGS grades. If the priority is to create a Pug-type dog with reduced respiratory compromise, crossbreeding between purebred Pugs and alternative longer-nosed breeds offers a potential route to breed dogs who are substantially less likely to suffer from the negative welfare impacts of increased respiratory compromise (e.g. unpleasant respiratory effort, and air hunger during exertion [Beausoleil & Mellor 2015]), by selecting towards less extreme conformations. The two Pug crosses also had a significantly lower median nostril grade (i.e. less stenotic nostrils) compared to the purebred Pugs, with nostril stenosis previously reported as a predictor for BOAS (Liu *et al.* 2017). Although stenotic nares were significantly less likely in the two Pug crosses, other abnormalities that could not be visualised (e.g. elongated soft palate) were not assessed in the veterinary examination. However, the increased relative muzzle length in the Pug crosses likely facilitates a functional airway anatomy that does not readily obstruct, as reflected by the majority scoring 0 on the RFGS.

The Royal Kennel Club updated their guidance in 2026 to recommend that dogs scored as Grade 3 or 2 on the RFGS are not used for breeding but was more conciliatory prior to this when dogs scored as Grade 2 were also included for breeding with advice on due consideration and care (Royal Kennel Club 2026). This brings the Royal Kennel Club’s breeding recommendations in relation to RFGS grades in line with a UK legal analysis from the Legal Advisory Group on Extreme Conformations in Dogs reports that “*Anyone responsible for a dog that scores Grade 2 or Grade 3 on the RFGS is on notice that this dog has clinically impaired respiratory function that is limiting the dog’s ability to perform their natural and legally protected behaviours. Any act (or failure to act) on the part of anyone responsible for a dog which further jeopardises the dog’s health and welfare arising from the existing impaired respiratory function would be liable to prosecution under the Animal Welfare Act (AWA)* 2006 *, potentially leading to a criminal conviction*” (LAGECDogs 2024). For commercial breeders, breeding from any dog which has scored a Grade 2 or 3 on the RFGS could therefore be considered an offence under the AWA, as both pregnancy and whelping are known to add further respiratory load to dogs with already impaired respiratory function (Lopate 2012). This understanding, paired with the results from the current study showing that 24/51 (47.1%) Pugs assessed scored a Grade 2 or 3, highlights the importance of informing breeders about their legal requirements to protect the welfare of breeding dogs and their progeny, and to urge breeders of extreme brachycephalics, such as Pugs, French Bulldogs and English Bulldogs to assess respiratory health before breeding. Based on results from the current study, almost half of UK purebred Pugs could be considered outside the realms of current UK legislation for commercial breeders to breed from. The UK Brachycephalic Working Group (BWG) has further stated that “*Noisy breathing in brachycephalic (flat-faced) dogs at rest or light exercise is never normal*” encouraging owners of a brachycephalic dog with noisy breathing to never breed from their dog, or to promote them as healthy (BWG 2024).

Results from the current study report significant variation in BCS between Pugs, Jugs and Puggles. Whilst 75% of Jugs had an ideal BCS score of 4–5/9, 38% of Puggles and 24% of Pugs were assessed as severely overweight with a BCS of 7–8/9. These results are perhaps unsurprising, as Pugs and Beagles have both been reported as breeds with increased odds for being overweight from UK veterinary practice data (Pegram *et al.* 2021), with obesity the most commonly recorded disorder for both Pugs (13.2–17.4% prevalence; O’Neill *et al.* 2016) and Beagles (24.3% prevalence; O’Neill *et al.* 2025). An increasing body of evidence points to genetics as an important contributor to canine obesity (Raffan 2016). Crossbreeding between two breeds with a propensity towards obesity appears to have exacerbated this predisposition in Puggles. Obesity carries additional concerns in brachycephalic breeds as an exacerbating factor for BOAS (Packer *et al.* 2015b; Liu *et al.* 2017). The high prevalence of over-ideal BCS in Puggles in the current study highlights the need for crossbreeding strategies to be carefully considered, to avoid perpetuating or even exacerbating parallel negative traits from both progenitor breeds (Bryson *et al.* 2024).

Pugs and Puggles were also found to have a significantly increased neck girth compared to Jugs, another conformational risk factor for BOAS (Packer *et al.* 2015b; Liu *et al.* 2017). The accumulation of conformational risk factors for BOAS in Pugs compared to Puggles and Jugs – higher BCS, increased neck girth, more stenotic nares and lower CFR –demonstrates the need to markedly alter the current conformation of Pugs to substantially reduce BOAS risks, and the opportunities afforded to do this via crossbreeding with carefully chosen non-brachycephalic breeds.

In the current study, Pugs had significantly increased owner-reported levels of ophthalmic disease compared to Jugs and Puggles. This is likely due to differences in conformation, with Pugs in the current study demonstrating significantly decreased CFR, increased likelihood of a nasal fold and increased exposed sclera compared to Jugs and Puggles. These features have been demonstrated to increase risk of corneal ulceration (Packer *et al.* 2015a), one of the most common ocular diseases seen in domestic dogs and particularly prevalent in Pugs (O’Neill *et al.* 2017a). Furthermore, purebred Pugs were more likely to be affected by entropion and ectropion than the two Pug crosses, with one in five Pugs affected by entropion. The 2020 Breed Health and Conservation Plan for Pugs includes eye disorders as a priority health issue for the breed (BWG 2021b). Without substantive conformation changes to Pugs’ craniofacial structure (including orbit depth), eyelid anatomy and facial skinfolds, it is unlikely that prevalence of conformation-related eye disorders can be substantially reduced. Crossbreeding offers a route towards rapid improvements in conformational risk factors associated with several eye disorders and consequently opportunities to markedly reduce prevalence.

In the current study, all purebred Pugs had a nasal fold, compared to around one in six Jugs and one in four Puggles. Pugs had a correspondingly significantly higher prevalence of nasal-fold dermatitis compared to the Pug-crosses (38.3% vs 0–12.5%). UK VetCompass data indicate that Pugs are one of the three most highly predisposed breeds to skin-fold dermatitis, alongside the English Bulldog and French Bulldog (O’Neill *et al.* 2022a). Given nasal folds are purely aesthetic and offer no welfare advantages to dogs, selection away from this harmful trait is urgently needed to reduce substantial risks to ocular and skin health (Packer *et al.* 2015a)., and the current study shows this can be rapidly achieved via crossbreeding.

Purebred Pugs showed substantially higher levels of visible signs of aural disease through scoring of stenosis, redness and discharge. Breed and ear carriage are reported as significant risk factors for aural disease, with brachycephalic breeds having increased odds of otitis externa compared to non-brachycephalic breeds (O’Neill *et al.* 2020). Beagles have also been identified as a breed with increased odds of ear disorders (O’Neill *et al.* 2021b); however, Puggles still scored lower than purebred Pugs in the current study. These results align with VetCompass data, where ophthalmic, dermatological and aural disorders were the top three group-level disorders in Pugs (O’Neill *et al.* 2016) and at a specific-disorder level Pugs have been reported with significantly increased odds of BOAS, stenotic nares, corneal ulceration, skin-fold dermatitis and aural discharge (O’Neill *et al.* 2022b). Owner-reported levels of ear disease did not vary significantly between Pugs and the two Pug-crosses, which may indicate that Pug owners are unaware of signs of ear disease in their dogs or, as seen more widely in the dog-owning population, underestimate the urgency that they require veterinary diagnosis and care (Farrow *et al.* 2026), which owners should be made aware of to ensure timely veterinary attention.

Pugs had significantly higher prevalence of patella luxation in the current study compared to the Pug-crosses. This concurs with previous research reporting that brachycephalic dogs have increased odds of patella luxation compared to non-brachycephalic breeds (O’Neill *et al.* 2020) and Pugs have increased odds compared to non-Pugs (O’Neill *et al.* 2022b). Postural and gait abnormalities were not significantly different between groups, and previous VetCompass research on Pug health did not report Pugs at higher odds of lameness compared to other breeds (O’Neill *et al.* 2022b). However, Pugs in the current study were significantly more likely to show reduced or absent paw placing, VT (visual and tactile) placing, hopping and wheelbarrow responses. Although Pug owners report lower levels of spinal malformations and intervertebral disc disease than other extreme brachycephalic breeds, such as French Bulldogs and English Bulldogs (Packer *et al.* 2019), vertebral malformations are commonly reported in brachycephalic breeds with screw-tails and can have varying levels of clinical neurological signs (Ryan *et al.* 2017). The 2020 Breed Health and Conservation Plan for Pugs includes spinal problems as a priority health issue for the breed (BWG 2021b), which likely requires conformational change away from screw tails to achieve significant improvements. To assist the population of breeders adhering to Royal Kennel Club breed standards to breed away from harmful malformations, revisions of breed standards are needed to avoid encouragement for a tail that is “*tightly curled*” (Royal Kennel Club 2025a) which currently is still included in the breed standard as a desirable feature.

Maxillary shortening in brachycephalic dogs often leads to rotational malocclusion, and dental crowding is also increased in small breed dogs due to proportionally larger teeth in relation to jaw size (Niemiec 2021). Pugs have been demonstrated to have higher odds of dental disorders than non-Pugs (O’Neill *et al.* 2022b). In the current study, owner-reporting of dental disease did not differ between breeds; although the direct veterinary examination during the practical health assessments in the study demonstrated significant differences in dental disorders in each of the three breed-types (e.g. crowding and missing/loose teeth were significantly more common in Pugs and Jugs compared to Puggles and Grade 1 malocclusions were most commonly observed in Pugs, while Grade 3 malocclusions were most commonly observed in Puggles). Crossbreeding therefore appears to have a mixed effect on dental health dependent on the non-brachycephalic breed involved in the cross, and does not necessarily eliminate conformational dental disorders, instead potentially redistributing the pattern of malocclusion rather than uniformly reducing pathology.

### Owner-reported health

Owner reports of brachycephaly-related dysfunction demonstrated significantly worse health in purebred Pugs compared to the two designer crosses across all domains studied (heat tolerance, eating, sleeping and breathing). Brachycephaly has been reported as a major risk factor for heat-related illness in dogs (Bruchim *et al.* 2006; Hall *et al.* 2020), with studies showing brachycephalic dogs have substantially decreased capacity for thermoregulation when exposed to higher temperatures than their non-brachycephalic counterparts (Davis *et al.* 2017). In the current study, Pug owners reported a significantly higher median heat-related dysfunction score compared to Jug and Puggle owners. Correspondingly, just under half (43.1%) of Pug owners reported their dog had a problem in the heat, significantly more than Jug (6.0%) and Puggle (12.5%) owners. Owners of purebred Pugs also reported higher scores for difficulties in their dogs associated with eating and sleeping than the owners of Jugs or Puggles. In dogs, sleeping and eating dysfunction have been linked to brachycephaly and BOAS (Poncet *et al.* 2005; Roedler *et al.* 2013; Niinikoski *et al.* 2024). For example, in a study of sleep-disordered breathing using neckband technology, brachycephaly was identified as a risk factor alongside obesity, with dogs with moderate-severe signs of BOAS a further risk factor (Niinikoski *et al.* 2024). Furthermore, the respiratory and gastrointestinal tracts are functionally interconnected through common mechanisms involved in breathing and swallowing, so pathology in one system can impact the other (Luciani *et al.* 2022). Results from the current study suggest that designer crossbreeds between a Pug progenitor and a non-brachycephalic progenitor have greater resilience than purebred Pugs to thermoregulation, sleep and eating disorders. This is likely due to restoration of a more functional airway and gastrointestinal anatomy due to the genetic contribution of their non-brachycephalic progenitor.

The ORB scores quantifying breathing dysfunctions revealed that 51% of Pugs in the current study scored above the threshold deemed to indicate clinically relevant airway disease in previous research (Packer *et al.* 2012). However, when asked directly, only 29.4% of Pug owners agreed their dog had a problem with breathing. These markedly different results suggest substantial normalisation of signs of respiratory compromise among Pug owners that reflect similar findings of other studies demonstrating this phenomenon. Previously, in a foundational study, 58% of owners of BOAS-affected dogs reported that their dog did not have a breathing problem, despite owners reporting a high frequency/severity of BOAS-related clinical signs in their dogs (Packer *et al.* 2012). More recently, in a sample of n = 2,168 extreme brachycephalic dogs (including n = 789 Pugs), nearly 40% of dogs reached an ORB score indicative of clinically relevant airway obstruction, while only 17.9% of owners of these dogs considered their dog to have a breathing problem (Packer *et al.* 2019). A similar cognitive dissonance effect was seen in a study of French Bulldogs, when comparison of owner perception of BOAS in their dog against RFGS grading revealed 60% of owners failed to recognise signs of respiratory compromise associated with BOAS in dogs who graded 2 or 3 (Liu *et al.* 2015). If many owners are consciously or subconsciously incapable of perceiving clinical signs as indicative of disease but are instead considering these signs as ‘normal for breed’, appropriate veterinary care may not be sought, and treatment may be delayed or absent completely (Packer *et al.* 2012). The normalisation of respiratory signs has been identified as most common in owners of extreme brachycephalic breeds who express a preference for flat faces and ‘laziness’ in dogs (Packer *et al.* 2024). Results from the current study indicate greater awareness amongst owners of brachycephalic designer crossbreeds, as 4.2% of Puggle owners considered their dog to have a breathing problem, and 4.2% of Puggles scored an 8 or over on the ORB score. For Jugs, 0% of owners agreed that their dog had a breathing problem, but only 2% (n = 1) of Jugs had a high enough ORB score to indicate clinically relevant disease. The more consistent results shown by owners of brachycephalic crossbreeds in the current study suggests that the widespread normalisation of signs of respiratory dysfunction (e.g. abnormal respiratory sounds) shown by owners of purebred Pugs may be restricted to those persons who have chosen to own a dog with an extreme conformation, and may not extend to owners of brachycephalic designer crossbreed dogs. Normalisation of clinical signs of conformation-related disorders in the purebred owner population may reflect strategies to reduce cognitive dissonance (Packer *et al.* 2019, 2024), to preserve the validity of their decision to acquire an extreme brachycephalic breed and to protect their personal identities as an owner of these breeds in the face of increasing information availability on the poor health of purebred Pugs that counters their own belief systems.

Purebred Pugs showed significantly higher mean scores for the Canine Brief Pain Inventory (CBPI) scale compared to Jugs and Puggles, and their overall quality of life was less likely to be rated by their owners as ‘excellent’. The CBPI is an owner-reported questionnaire which asks owners to rate their dogs’ pain, and to rate their dogs’ activity level and quality of life. The CBPI has been used to assess both osteoarthritic pain and cancer pain in dogs (Brown *et al.* 2007, 2008, 2009). Chronic pain can impact cognitive and emotional health, resulting in poor welfare states (Malkani *et al.* 2024). Sources of pain in the purebred Pug population may originate from painful disorders that the breed is predisposed to, e.g. musculoskeletal pain from patella luxation or aural pain from otitis externa (O’Neill *et al.* 2022b), and which were also more common in Pugs compared to Jugs or Puggles in the current study. Dog owners have been considered the animal’s proxy in the assessment of canine chronic pain (Reid *et al.* 2018). However, decreased facial expressions have been demonstrated in quantitative analyses of brachycephalic dogs compared to normocephalic dogs (Martvel *et al.* 2025), which may disrupt the communicative content of animals’ faces and hinder owner detection of pain in their dogs, as demonstrated in brachycephalic cats (Finka *et al.* 2020), and thus the CBPI scores reported here may even be underestimates of the true scale of the issue.

### Financial implications

The increased disease burden in purebred Pugs compared to the two designer crossbreeds was reflected in their substantially higher veterinary costs. Purebred Pugs had a median monthly estimated spend on veterinary care of £41.67 (annual: £500.04), that was significantly higher than in Jugs (monthly £9.65; annual £115.80) and Puggles (monthly £10.17; annual £122.04). These costs are higher than previously reported for brachycephalic dogs, where the median owner-reported veterinary cost per year of ownership for Pugs, Bulldogs and French Bulldogs was £222.22 per year (~£18.51 per month) (Packer *et al.* 2019). This difference may reflect soaring veterinary costs in recent years (Zhang *et al.* 2024), some of which has been fuelled by substantial growth in the range and provision of advanced veterinary care for companion animals (Stull *et al.* 2018), that is likely facilitated by modern owner expectations that pets should have access to the same treatment options that are available to human patients (Corr *et al.* 2024). Despite higher veterinary costs for purebred Pugs, those owners did not seem overly surprised by this finding, with only 27.5% of purebred Pug owners stating that their veterinary costs had been above expectations. Violations of financial expectations were substantially lower in this study population of Pug owners compared to the 45% of dog owners (of all breeds and crossbreeds) who underestimated the minimum monthly cost of owning a pet reported by the 2023 PDSA Animal Wellbeing Report (PDSA 2023). The high financial cost of Pug veterinary care therefore seems to be an expected and accepted consequence of ownership, rather than a barrier. This aligns with previous studies reporting cost not to be a significant factor in acquisition choice for brachycephalic breeds (Packer *et al.* 2017), and that only 25.6% of current owners of extreme brachycephalic breeds consider veterinary costs a reason against acquiring a brachycephalic breed, compared to 36.9% of owners of non-brachycephalic dogs (Packer *et al.* 2024). This is also reflected in significantly fewer Pug owners reported lower than expected veterinary costs (9.8%) in comparison to Jug (23.5%) and Puggle owners (29.8%). As such, campaigning focused on the financial implications of Pug ownership are unlikely to perturb many owners from acquiring this breed.

In the current study, dog purchase costs also varied significantly between the purebred Pugs and the crossbreed Jugs and Puggles, with the median cost of Pugs £1,075, Jugs £500 and Puggles £600. Previous studies found the median purchase cost across multiple dog breeds as £600 – most comparable with the crossbreed dogs in the current study – and also reported higher purchase costs for purebred brachycephalic dogs (median £1,200) (Packer *et al.* 2017). Furthermore, a study of online advertisements of puppies reported that dogs from breeds with extreme conformation were priced at £208.07 more expensive, on average, than breeds without extreme conformation (Ross *et al.* 2023). The relatively high purchase cost for the purebred Pugs may reflect ongoing public perception of the desirability of owning pedigree dogs (Bir *et al.* 2017; Strand *et al.* 2019), particularly those with extreme conformation.

### Behaviour and dog-owner relationships

In the current study, the purebred Pugs displayed lower scores for trainability, in line with previous studies reporting that brachycephalic dogs are perceived to be less trainable than mesocephalic dogs (Helton 2009). Higher trainability scores were seen for Jugs and Puggles compared to Pugs in the current study. This counters previously documented perceptions that brachycephalic crossbreed dogs are less trainable than purebred brachycephalic dogs, cited as a negative consequence of crossbreeding purebred brachycephalic breeds with other purebred non-brachycephalic breed (Youens *et al.* 2026). In the current study, scores for behavioural traits (via the C-BARQ), emotional closeness with their owner and perceived costs of ownership (via the MDORS) were generally similar between all three breeds. Perceived positive behavioural traits, such as being a good companion and being good with children, have previously been reported as strongly influential in the acquisition of Pugs, English Bulldogs and French Bulldogs, as well as a reason why they would recommend their breed to others (Packer *et al.* 2020). This is despite a lack of evidence of a predisposition towards these traits (Packer *et al.* 2017). As such, the findings of this study can contribute to a stronger evidence base showing that Jug and Puggle brachycephalic crossbreeds retain the positive temperament and dog-owner relationships of their purebred brachycephalic progenitors, and that purebred brachycephalic breeds uniquely possess these desired traits (Packer *et al.* 2020).

### Study limitations

The owners in this study were predominantly female, which follows known trends in online sample selection (Curtin *et al.* 2000). However, owner demographics did not vary significantly between the three breeds. Dogs from a range of ages (1.3–8.5 years) were recruited with no significant age variation between breeds. However, it is possible that the proportional health reported in the current results may differ between the three breeds beyond this age, although it should be noted that the life expectancy of Pugs is shortened compared to the general dog population, at 7.65 years (6.99–8.20) (Teng *et al.* 2022). Results from the current study are limited to Pugs, Jugs and Puggles in the UK and therefore should be generalised with caution to more global populations and other brachycephalic breeds and designer crossbreeds. However, the core biological principles of moderating conformation via crossbreeding between brachycephalic and normocephalic breeds are likely to apply widely given shared risk factors for disease across brachycephalic breeds. Although both RKC-registered and non-registered Pugs were included in this study, 35.3% of studied Pugs were registered, mirroring (and slightly exceeding) the percentage of UK dogs registered (~25%), demonstrating our sample is representative of the UK Pug population. The RFGS is currently validated for 17 purebred brachycephalic breeds, including the Pug, and has not been evaluated for use in crossbreed dogs. Despite this, it is reasonable to assume that it would provide relevant information in this context, as a more structured version of a veterinary examination of respiratory dysfunction conducted by veterinary surgeons in practice to diagnose BOAS. All assessments were undertaken by a single examiner (veterinary surgeon EY) who was qualified to carry out RFGS assessments to avoid the issue of inter-rater reliability which has been demonstrated as potentially problematic in the scheme (Peng *et al.* 2025). The current study also relies partially on owner-reported health data, but the coupling of owner-reported disorders with practical veterinary assessment was used to reduce bias. Reassuringly, owner and practical reports largely agreed – and where they disagreed, known influences (e.g. normalisation of clinical signs by owners) were likely a major contributor to disparities.

### Animal welfare implications

The increasing extremeness of dog conformation at a population level is of high concern to those involved in canine welfare. In the current study, we show that brachycephalic crossbreed dogs fulfil many of the ownership desires that are currently fulfilled by extreme brachycephalic purebreds, whilst dramatically reducing health and welfare issues, often in just one generation. This offers an evidence-based strategy to the breeding community to rapidly reverse many of the harmful effects of extreme brachycephaly and therefore to improve canine welfare.

## Conclusion

The current study demonstrates that crossbreeding between an extreme brachycephalic breed (Pug) and one of two normocephalic, moderate breeds (Jack Russell Terrier and Beagle) can result in a new type of dog that is less physically extreme than the extreme progenitor. Crossbreeding resulted in substantially reduced risks of a range of disorders linked to extreme conformation in the (often first generation) offspring compared to the extreme purebred progenitor. Compared to purebred Pugs, the crossbred Jugs and Puggles exhibited markedly reduced prevalence of welfare-priority and highly predisposed disorders in the Pug, including respiratory, ocular, dermatological and musculoskeletal disorders. Conversely, the designer crossbreed dogs retained similar behavioural characteristics and strong dog-owner attachment seen in purebred Pugs which are strong motivators for their acquisition. These results counter many of the commonly cited concerns regarding the crossbreeding of extreme brachycephalic dogs with non-extreme breeds and provide strong evidence to support future international initiatives to use this crossbreeding as a powerful tool to reduce the currently serious health and welfare issues of extreme brachycephalic breeds, in a rapid and effective manner.

## Supporting information

10.1017/awf.2026.10095.sm001 Youens et al. supplementary material Youens et al. supplementary material

## Supplementary material

The supplementary material for this article can be found at http://doi.org/10.1017/awf.2026.10095.

## Long descriptions

### Table 1. Long description

The table consists of seven columns: Domain, Rating, Pug (n = 51), Jug (n = 51), Puggle (n = 48), X-squared, and P-value.

1. Veterinary costs: P-value is 0.008. Below expectations: Pug 5 (9.8 percent), Jug 12 (23.5 percent), Puggle 14 (29.8 percent). Met expectations: Pug 32 (62.7 percent), Jug 34 (66.7 percent), Puggle 30 (62.5 percent). Above expectations: Pug 14 (27.5 percent), Jug 5 (9.8 percent), Puggle 3 (6.4 percent).

2. Exercise: P-value is less than 0.001. Below expectations: Pug 9 (17.6 percent), Jug 0 (0.0 percent), Puggle 0 (0.0 percent). Met expectations: Pug 30 (58.8 percent), Jug 33 (64.7 percent), Puggle 36 (76.6 percent). Above expectations: Pug 12 (23.5 percent), Jug 18 (35.3 percent), Puggle 11 (23.4 percent).

3. Behaviour: P-value is 0.039. Below expectations: Pug 4 (7.8 percent), Jug 4 (7.8 percent), Puggle 7 (14.9 percent). Met expectations: Pug 36 (70.6 percent), Jug 45 (88.2 percent), Puggle 36 (76.6 percent). Above expectations: Pug 11 (21.6 percent), Jug 2 (3.9 percent), Puggle 4 (8.5 percent).

4. Maintenance: P-value is 0.053. Below expectations: Pug 3 (5.9 percent), Jug 1 (2.0 percent), Puggle 2 (4.3 percent). Met expectations: Pug 40 (78.4 percent), Jug 49 (96.1 percent), Puggle 43 (91.5 percent). Above expectations: Pug 8 (15.7 percent), Jug 1 (2.0 percent), Puggle 2 (4.3 percent).

Navigate back to Table 1..

### Table 2. Long description

The table consists of six columns: Reported health problem, Pug n equals 51, Jug n equals 51, Puggle n equals 48, X super 2, and P-value.

* Dental: Pug 11 21.6 percent, Jug 10 19.6 percent, Puggle 9 18.8 percent, X super 2 0.130, P-value 0.937.

* Orthopaedic: Pug 3 5.9 percent, Jug 3 5.9 percent, Puggle 6 12.5 percent, X super 2 1.942, P-value 0.379.

* Ocular: Pug 18 35.3 percent, Jug 3 5.9 percent, Puggle 6 12.5 percent, X super 2 17.466, P-value less than 0.001.

* Skin: Pug 5 9.8 percent, Jug 1 2.0 percent, Puggle 4 8.3 percent, X super 2 2.836, P-value 0.242.

* Ears: Pug 3 5.9 percent, Jug 5 9.8 percent, Puggle 5 10.4 percent, X super 2 0.767, P-value 0.681.

* Respiratory: Pug 11 21.6 percent, Jug 0 0.0 percent, Puggle 0 0.0 percent, X super 2 23.043, P-value less than 0.001.

Statistically significant P-values are found for Ocular and Respiratory problems.

Navigate back to Table 2..

### Table 3. Long description

The table consists of six columns: Owner reported dysfunction score, Pug n equals 51, Jug n equals 51, Puggle n equals 48, Kruskal-Wallis, and P-value.

* Heat domain (out of 12): Pugs have a median of 4.0 (range 0 to 8); Jugs have 2.0 (range 0 to 6); Puggles have 2.0 (range 0 to 8). Kruskal-Wallis is 24.42 with a P-value less than 0.001.

* Eating domain (out of 20): Pugs have a median of 3.0 (range 0 to 11); Jugs have 0.0 (range 0 to 6); Puggles have 1.0 (range 0 to 5). Kruskal-Wallis is 19.54 with a P-value less than 0.001.

* Sleeping domain (out of 30): Pugs have a median of 6.0 (range 0 to 18); Jugs have 2.0 (range 0 to 11); Puggles have 1.0 (range 0 to 7). Kruskal-Wallis is 29.23 with a P-value less than 0.001.

* Breathing O R B domain (out of 40): Pugs have a median of 8.0 (range 0 to 22); Jugs have 2.0 (range 0 to 10); Puggles have 2.0 (range 0 to 10). Kruskal-Wallis is 42.22 with a P-value less than 0.001.

All P-values are statistically significant as indicated by bold text in the original data.

Navigate back to Table 3..

### Table 4. Long description

The table contains six columns: C B A R Q scale, Pug median score (n equals 51), Jug median score (n equals 51), Puggle median score (n equals 48), Kruskal-Wallis, and P-value.

* Excitability: Pug 7.00, Jug 7.00, Puggle 6.00, Kruskal-Wallis 2.715, P-value 0.257.

* Aggression: Pug 5.00, Jug 6.00, Puggle 8.00, Kruskal-Wallis 5.340, P-value 0.070.

* Fear and anxiety: Pug 8.00, Jug 10.00, Puggle 8.00, Kruskal-Wallis 0.084, P-value 0.959.

* Separation: Pug 2.00, Jug 2.00, Puggle 1.00, Kruskal-Wallis 3.149, P-value 0.207.

* Attachment and attention-seeking: Pug 7.00, Jug 7.00, Puggle 6.00, Kruskal-Wallis 8.890, P-value 0.012 (statistically significant).

* Trainability: Pug 7.00, Jug 8.00, Puggle 8.00, Kruskal-Wallis 9.929, P-value 0.007 (statistically significant).

Navigate back to Table 4..

### Table 5. Long description

The table contains six columns: Body measurement, Pug mean plus or minus S D value n equals 51, Jug mean plus or minus S D value n equals 51, Puggle mean plus or minus S D value n equals 48, F-statistic, and P-value. All P-values are statistically significant.

* Muzzle length in mm: Pug 15.53 plus or minus 4.94, Jug 39.39 plus or minus 7.55, Puggle 40.06 plus or minus 7.69, F 211.768, P less than 0.001.

* Cranial length in mm: Pug 104.20 plus or minus 13.36, Jug 105.59 plus or minus 11.44, Puggle 111.65 plus or minus 14.64, F 4.426, P 0.014.

* Craniofacial ratio C F R: Pug 0.15 plus or minus 0.05, Jug 0.38 plus or minus 0.09, Puggle 0.36 plus or minus 0.06, F 161.527, P less than 0.001.

* Eye width in mm: Pug 55.88 plus or minus 8.32, Jug 42.63 plus or minus 7.36, Puggle 46.31 plus or minus 7.29, F 40.406, P less than 0.001.

* Palpebral fissure in mm: Pug 26.32 plus or minus 4.50, Jug 28.65 plus or minus 4.00, Puggle 34.23 plus or minus 5.03, F 39.454, P less than 0.001.

* Relative palpebral fissure width: Pug 25.25 plus or minus 3.38, Jug 27.39 plus or minus 3.54, Puggle 30.84 plus or minus 3.98, F 10.921, P 0.006.

* Head width in mm: Pug 123.76 plus or minus 11.61, Jug 121.18 plus or minus 13.48, Puggle 137.92 plus or minus 23.34, F 14.12, P less than 0.001.

* Neck length in mm: Pug 151.71 plus or minus 21.58, Jug 151.27 plus or minus 23.08, Puggle 186.21 plus or minus 30.20, F 31.178, P less than 0.001.

* Neck girth in mm: Pug 359.00 plus or minus 30.79, Jug 316.47 plus or minus 25.75, Puggle 368.96 plus or minus 49.54, F 29.24, P less than 0.001.

* Chest girth in mm: Pug 522.31 plus or minus 52.06, Jug 496.78 plus or minus 40.92, Puggle 610.71 plus or minus 90.87, F 42.438, P less than 0.001.

* Body length in mm: Pug 351.27 plus or minus 37.07, Jug 337.53 plus or minus 22.89, Puggle 410.46 plus or minus 52.07, F 48.673, P less than 0.001.

* Height at withers in mm: Pug 320.88 plus or minus 28.10, Jug 340.29 plus or minus 38.48, Puggle 372.83 plus or minus 48.07, F 22.457, P less than 0.001.

* Height at tail-base in mm: Pug 313.08 plus or minus 29.23, Jug 335.69 plus or minus 5.45, Puggle 374.17 plus or minus 47.28, F 30.908, P less than 0.001.

Navigate back to Table 5..

### Table 6. Long description

A table presenting logistic regression results for R F G grade 0 or 1 versus grade 2 or 3. The table is divided into two main sections: Model 1 C F R and Model 2 Breed. Each model reports O R, 95 percent C I, and P-value.

* Intercept: Model 1 P-value 0.995; Model 2 P-value 0.00.

* C F R (Continuous): Model 1 O R not listed, 95 percent C I 0.00 to 0.02, P-value 0.018. Model 2 data is not applicable.

* Breed: Jug is the base category for both models. For Model 2, Pug has an O R of 38.5, 95 percent C I 3.66 to 534.44, and P-value less than 0.001. Puggle has an O R of 0.57, 95 percent C I 0.03 to 11.01, and P-value 0.786.

* Age (months): Model 1 O R 1.07, P-value 0.745. Model 2 O R 1.11, P-value 0.518.

* Sex: Female entire is the base category. For Model 1, Male entire shows the highest O R at 29.00 (P-value 0.072). For Model 2, Male entire O R is 6.94 (P-value 0.133).

* B C S (Continuous): Model 1 O R 2.29, P-value 0.035. Model 2 O R 1.76, P-value 0.061.

* Nostril grade (Continuous): Model 1 O R 1.97, P-value 0.400. Model 2 O R 1.71, P-value 0.356.

Navigate back to Table 6..

### Table 7. Long description

The table consists of six columns: Skin fold location, Pug n equals 51, Jug n equals 51, Puggle n equals 48, X-squared, and P-value.

Row 1: Nasal fold. Pug 51 100.0 percent, Jug 8 15.7 percent, Puggle 13 27.1 percent, X-squared 84.999, P-value less than 0.001.

Row 2: Nasal fold dermatitis. Pug 18 38.3 percent, Jug 1 2.0 percent, Puggle 0 0.0 percent, X-squared 8.026, P-value 0.018.

Row 3: Tail fold. Pug 10 19.6 percent, Jug 6 11.8 percent, Puggle 3 6.3 percent, X-squared 4.045, P-value 0.132.

Row 4: Tail fold dermatitis. Pug 4 7.8 percent, Jug 2 3.9 percent, Puggle 1 2.1 percent, X-squared 0.090, P-value 0.956.

Row 5: Other skin folds. Pug 22 43.1 percent, Jug 5 9.8 percent, Puggle 4 8.3 percent, X-squared 23.830, P-value less than 0.001.

Row 6: Other skin fold dermatitis. Pug 6 11.8 percent, Jug 0 0.0 percent, Puggle 1 2.1 percent, X-squared 1.968, P-value 0.374.

Statistically significant P-values are found for Nasal fold, Nasal fold dermatitis, and Other skin folds.

Navigate back to Table 7..

### Table 8. Long description

The table compares dental abnormalities across three groups: Pug (n = 51), Jug (n = 51), and Puggle (n = 48). Columns include the abnormality, breed percentages, Chi-squared (X super 2) values, and P-values.

* Halitosis: Pug 41.2 percent, Jug 17.6 percent, Puggle 35.4 percent. P-value 0.029 (significant).

* Calculus: Distributed across Grades 0 to 2. Grade 1 is most common in Pugs (51.0 percent) and Puggles (50.0 percent). P-value 0.226.

* Gingivitis: Distributed across Grades 0 to 3. Grade 1 is most common in Pugs (66.7 percent). P-value 0.006 (significant).

* Missing or loose teeth: Pug 51.0 percent, Jug 54.9 percent, Puggle 31.3 percent. P-value 0.042 (significant).

* Retained deciduous teeth: Pug 19.6 percent, Jug 13.7 percent, Puggle 6.3 percent. P-value 0.147.

* Dental crowding: Pug 72.5 percent, Jug 78.4 percent, Puggle 56.3 percent. P-value 0.047 (significant).

* Malocclusion: Distributed across Grades 0 to 4. Grade 3 is the most frequent across all breeds: Pug 49.0 percent, Jug 76.5 percent, and Puggle 68.8 percent. P-value less than 0.001 (highly significant).

Navigate back to Table 8..

### Table 9. Long description

A table with six columns: Orthopaedic abnormality, Pug percentage (n equals 51), Jug percentage (n equals 51), Puggle percentage (n equals 48), X super 2, and P-value.

Data rows include:

* Abnormal posture: Pug 1 (2.0 percent), Jug 0 (0.0 percent), Puggle 0 (0.0 percent), X super 2 1.954, P-value 0.376.

* Gait lame: Pug 5 (9.8 percent), Jug 3 (5.9 percent), Puggle 5 (10.4 percent), X super 2 0.769, P-value 0.681.

* Gait ataxic: Pug 2 (3.9 percent), Jug 0 (0.0 percent), Puggle 2 (4.2 percent), X super 2 2.123, P-value 0.346.

* Gait scuffing: Pug 3 (5.9 percent), Jug 0 (0.0 percent), Puggle 0 (0.0 percent), X super 2 5.942, P-value 0.051.

* Paw placing: Pug 7 (13.7 percent), Jug 0 (0.0 percent), Puggle 2 (4.2 percent), X super 2 8.851, P-value 0.012.

* Visual / tactile placing: Pug 6 (11.8 percent), Jug 0 (0.0 percent), Puggle 1 (2.1 percent), X super 2 8.965, P-value 0.011.

* Hopping test: Pug 10 (19.6 percent), Jug 1 (2.0 percent), Puggle 2 (4.2 percent), X super 2 11.838, P-value 0.003.

* Wheelbarrow test: Pug 7 (13.7 percent), Jug 0 (0.0 percent), Puggle 2 (4.2 percent), X super 2 10.241, P-value 0.006.

* Reduced muscle: Pug 14 (27.5 percent), Jug 3 (5.9 percent), Puggle 2 (4.2 percent), X super 2 17.807, P-value less than 0.001.

* Reduced flexibility: Pug 22 (43.1 percent), Jug 6 (11.8 percent), Puggle 13 (27.1 percent), X super 2 12.638, P-value 0.002.

* Patella luxation: Pug 9 (17.6 percent), Jug 3 (5.9 percent), Puggle 2 (4.2 percent), X super 2 6.398, P-value 0.041.

Navigate back to Table 9..
