# Supplementary material for: A solution right under our nose? Crossbreeding extreme brachycephalic dogs results in better health outcomes while preserving behavioural profiles and dog-owner relationship quality
Source: Anim Welf. 2026 Aug 5;35:e41. doi: 10.1017/awf.2026.10095 (PMC13443976; doi:10.1017/awf.2026.10095)
Supplement: Supplementary file 1 [file S0962728626100955sup001.pdf]

A solution right under our nose? Crossbreeding extreme brachycephalic dogs results in better health outcomes while preserving behavioural profiles and dog-owner relationship quality: Supplementary material

Elizabeth Youens[1], Dan G O'Neill[2], Michael S Tivers[3], Zoe Belshaw[4], Johanna Neufuss[5], Rowena MA Packerhttps://orcid.org/0000-0002-9988-9828[1]

[1] Clinical Science and Services, Royal Veterinary College, Herts, UK

[2] Pathobiology and Population Sciences, Royal Veterinary College, Herts, UK

[3] Paragon Veterinary Referrals, Wakefield, UK

[4] EviVet Evidence-Based Veterinary Consultancy, Nottingham, UK

[5] Blue Cross, Burford, UK

Author for correspondence: Rowena MA Packer, email: rpacker@rvc.ac.uk

S1 – Study advertising posters

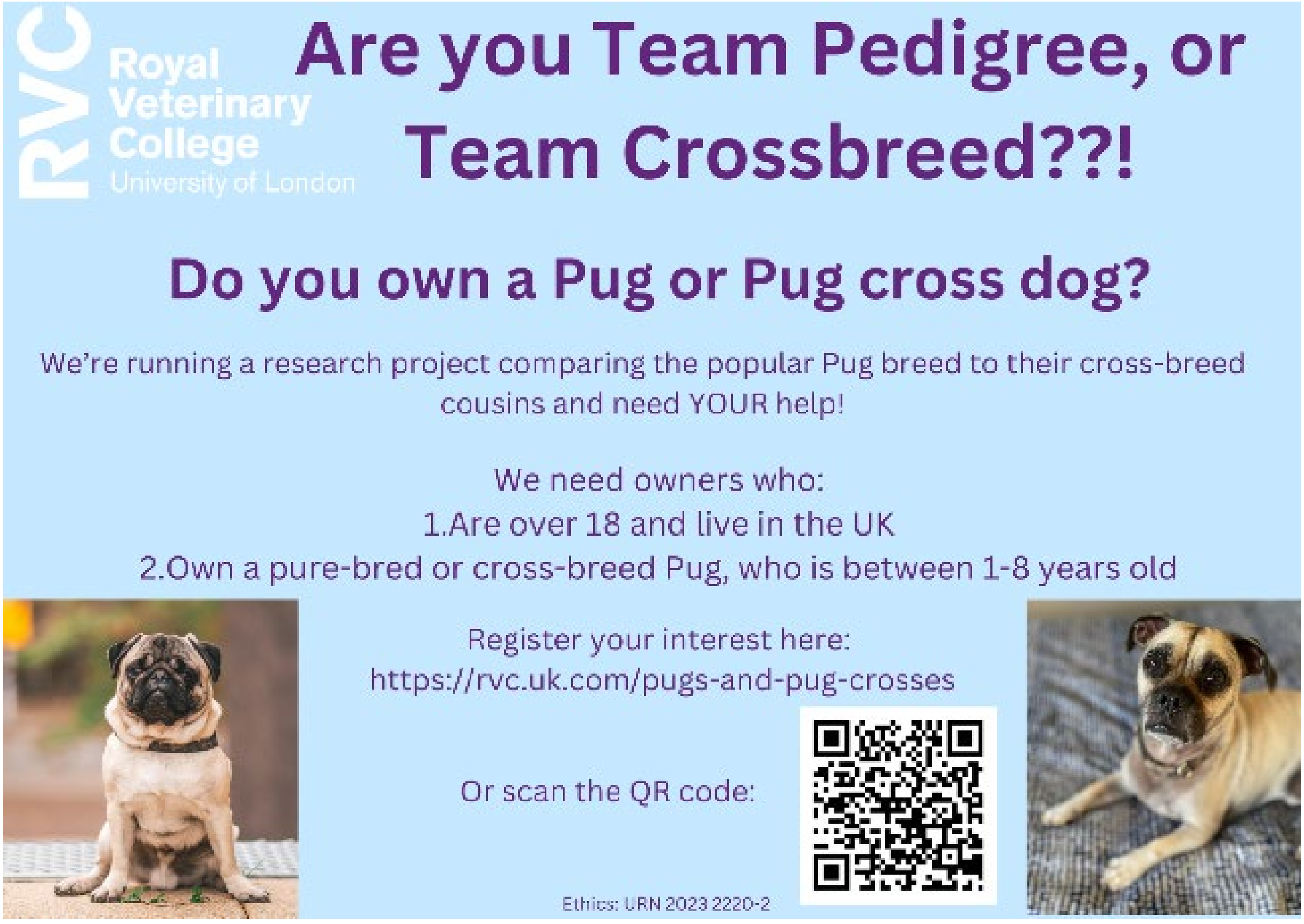

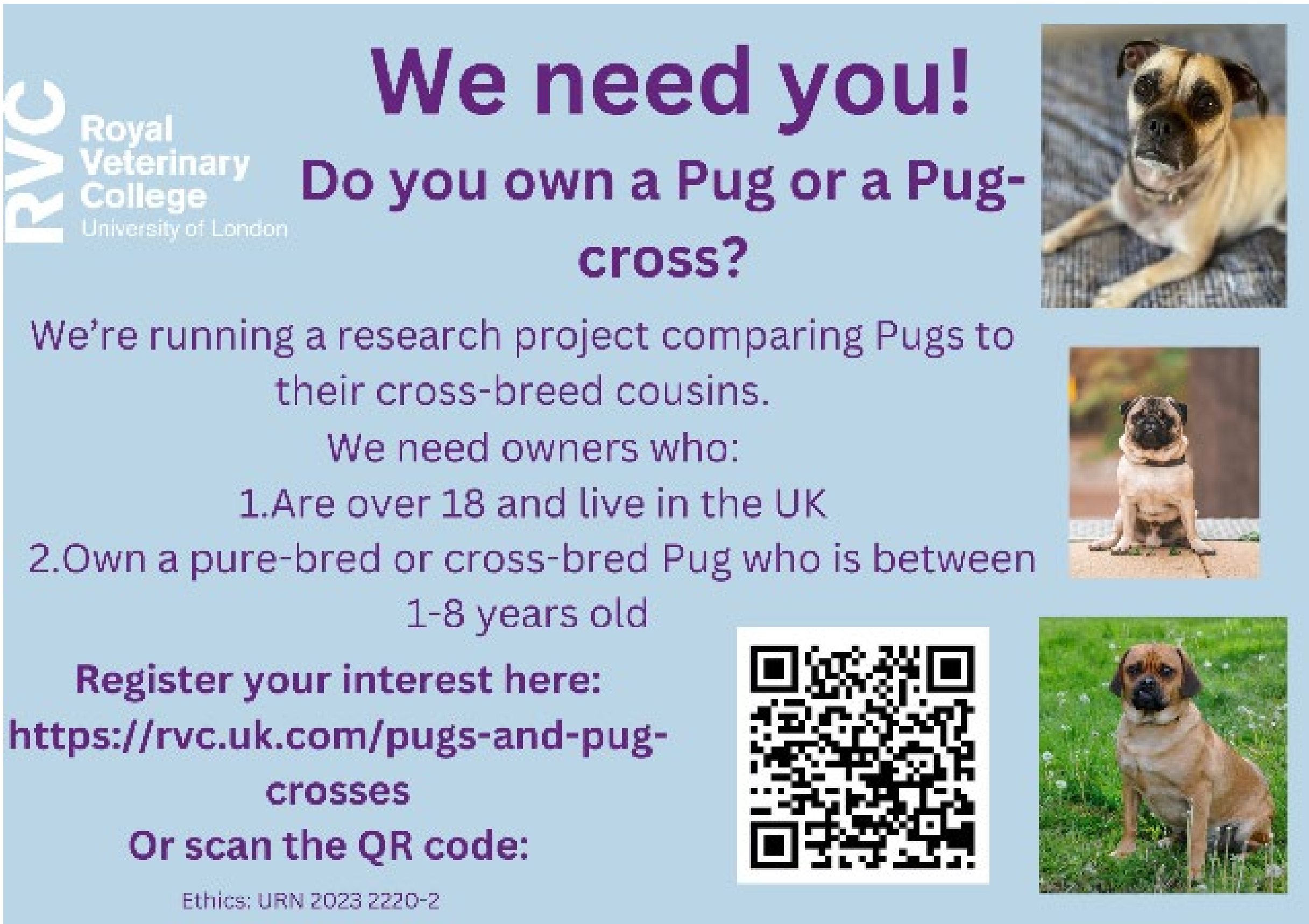

S2 – Disorder scoring system

When your dog interacts with other dogs or people, do you see them					
**Sign**	**Never**	**Rarely**	**Sometimes**	**Usually**	**Always**
Wag tail	0	1	2	3	4
Raise tail high	0	1	2	3	4
Tuck tail between legs	0	1	2	3	4
Prick ears forwards	0	1	2	3	4
Pin ears back	0	1	2	3	4

In warm weather, how often does your dog show the following behaviours?					
**Sign**	**Never**	**Rarely**	**Sometimes**	**Usually**	**Always**
Heavy panting	0	1	2	3	4
Laboured breathing	0	1	2	3	4
Collapse	0	1	2	3	4

While or after eating, how often does your dog show the following behaviour?						
**Sign**	**Never**	**Rarely**	**Monthly**	**Weekly**	**Daily**	**> Once per day**
Choking or gagging	0	1	2	3	4	5
Laboured breathing	0	1	2	3	4	5
Regurgitation	0	1	2	3	4	5
Vomiting	0	1	2	3	4	5

While sleeping, how often does your dog show the following behaviours?						
**Sign**	**Never**	**Rarely**	**Monthly**	**Weekly**	**Daily**	**> Once per day**
Open mouth	0	1	2	3	4	5
Sitting position	0	1	2	3	4	5
Head in elevated position	0	1	2	3	4	5
With a toy in their mouth	0	1	2	3	4	5
Stops breathing momentarily	0	1	2	3	4	5
Changes positions frequently	0	1	2	3	4	5

How often does your dog show difficulty breathing* in the following situations?						
	**Never**	**Rarely**	**Monthly**	**Weekly**	**Daily**	**> Once a day**
At rest (e.g. lying down but awake)	0	1	2	3	4	5
Gently walking (e.g. around house)	0	1	2	3	4	5
Exercise or activity	0	1	2	3	4	5
Asleep	0	1	2	3	4	5

*difficulty breathing = appearing short of breath, cannot keep up with you, can seem anxious

What does your dog's breathing sound like in the following situations?						
	**Very quiet**	**Panting but no snoring/ snorting/ wheezing**	**Slight snorting/ snoring/ wheezing**	**Some snorting/ snoring/ wheezing**	**Frequent snoring/ snorting/ wheezing**	**Almost continuous snoring/ snorting/ wheezing**
At rest (e.g. lying down but awake)	0	1	2	3	4	5
Gently walking (e.g. around house)	0	1	2	3	4	5
Exercise or activity	0	1	2	3	4	5
Asleep	0	1	2	3	4	5
